# Psychosis brain subtypes validated in first-episode cohorts and related to illness remission: results from the PHENOM consortium

**DOI:** 10.1038/s41380-023-02069-0

**Published:** 2023-05-05

**Authors:** Dominic B. Dwyer, Ganesh B. Chand, Alessandro Pigoni, Adyasha Khuntia, Junhao Wen, Mathilde Antoniades, Gyujoon Hwang, Guray Erus, Jimit Doshi, Dhivya Srinivasan, Erdem Varol, Rene S. Kahn, Hugo G. Schnack, Eva Meisenzahl, Stephen J. Wood, Chuanjun Zhuo, Aristeidis Sotiras, Russell T. Shinohara, Haochang Shou, Yong Fan, Maristela Schaulfelberger, Pedro Rosa, Paris A. Lalousis, Rachel Upthegrove, Antonia N. Kaczkurkin, Tyler M. Moore, Barnaby Nelson, Raquel E. Gur, Ruben C. Gur, Marylyn D. Ritchie, Theodore D. Satterthwaite, Robin M. Murray, Marta Di Forti, Simone Ciufolini, Marcus V. Zanetti, Daniel H. Wolf, Christos Pantelis, Benedicto Crespo-Facorro, Geraldo F. Busatto, Christos Davatzikos, Nikolaos Koutsouleris, Paola Dazzan

**Affiliations:** 1 Department of Psychiatry and Psychotherapy, Ludwig-Maximilian University, Munich, Germany; 2 Centre for Youth Mental Health, University of Melbourne, Melbourne, VIC Australia; 3 Orygen, Melbourne, VIC Australia; 4 Center for Biomedical Image Computing and Analytics, Perelman School of Medicine, University of Pennsylvania, Philadelphia, PA USA; 5 Department of Radiology, School of Medicine, Washington University in St. Louis, St. Louis, MO USA; 6 Department of Neurosciences and Mental Health, Fondazione IRCCS Ca’ Granda Ospedale Maggiore Policlinico, Milan, Italy; 7 Social and Affective Neuroscience Group, MoMiLab, IMT School for Advanced Studies Lucca, Lucca, Italy; 8 Max-Planck Institute of Psychiatry, Munich, Germany; 9 Department of Statistics, Zuckerman Institute, Columbia University, New York, NY USA; 10 Department of Psychiatry, Icahn School of Medicine at Mount Sinai, New York, NY USA; 11 Department of Psychiatry, University Medical Center Utrecht, Utrecht, Netherlands; 12 LVR-Klinikum Düsseldorf, Kliniken der Heinrich-Heine-Universität, Düsseldorf, Germany; 13 University of Birmingham, Edgbaston, UK; 14 Department of Psychiatric-Neuroimaging-Genetics and Co-morbidity Laboratory (PNGC-Lab), Nankai University Affiliated Tianjin Anding Hospital; Department of Psychiatry, Tianjin Medical University, Tianjin, China; 15 Department of Radiology and Institute for Informatics, Washington University School of Medicine, Washington University in St. Louis, St. Louis, MO USA; 16 Department of Biostatistics, Epidemiology, and Informatics, Perelman School of Medicine, University of Pennsylvania, Philadelphia, PA USA; 17 Institute of Psychiatry, Faculty of Medicine, University of São Paulo, São Paulo, Brazil; 18 Institute for Mental Health and Centre for Brain Health, University of Birmingham, Birmingham, UK; 19 Early Intervention Service, Birmingham Women’s and Children’s NHS Foundation Trust, Birmingham, UK; 20 Department of Psychology, Vanderbilt University, Nashville, TN USA; 21 Department of Psychiatry, Perelman School of Medicine, University of Pennsylvania, Philadelphia, PA USA; 22 Department of Genetics, Perelman School of Medicine, University of Pennsylvania, Philadelphia, PA USA; 23 Penn Lifespan Informatics and Neuroimaging Center, University of Pennsylvania, Philadelphia, PA USA; 24 Institute of Psychiatry, Psychology, and Neuroscience, King’s College London, London, UK; 25 Hospital Sírio-Libanês, São Paulo, Brazil; 26 Melbourne Neuropsychiatry Centre, Department of Psychiatry, University of Melbourne and Melbourne Health, Carlton South, VIC Australia; 27 Mental Health Service, Hospital Universitario Virgen del Rocío, Seville, Spain; 28 Centro de Investigación Biomédica en Red de Salud Mental, Instituto de Salud Carlos III (CIBERSAM), Madrid, Spain; 29 Instituto de Biomedicina de Sevilla (IBiS), Seville, Spain; 30 Department of Psychiatry, Universidad de Sevilla, Seville, Spain

**Keywords:** Neuroscience, Diagnostic markers

## Abstract

Using machine learning, we recently decomposed the neuroanatomical heterogeneity of established schizophrenia to discover two volumetric subgroups—a ‘lower brain volume’ subgroup (SG1) and an ‘higher striatal volume’ subgroup (SG2) with otherwise normal brain structure. In this study, we investigated whether the MRI signatures of these subgroups were also already present at the time of the first-episode of psychosis (FEP) and whether they were related to clinical presentation and clinical remission over 1-, 3-, and 5-years. We included 572 FEP and 424 healthy controls (HC) from 4 sites (Sao Paulo, Santander, London, Melbourne) of the PHENOM consortium. Our prior MRI subgrouping models (671 participants; USA, Germany, and China) were applied to both FEP and HC. Participants were assigned into 1 of 4 categories: subgroup 1 (SG1), subgroup 2 (SG2), no subgroup membership (‘None’), and mixed SG1 + SG2 subgroups (‘Mixed’). Voxel-wise analyses characterized SG1 and SG2 subgroups. Supervised machine learning analyses characterized baseline and remission signatures related to SG1 and SG2 membership. The two dominant patterns of ‘lower brain volume’ in SG1 and ‘higher striatal volume’ (with otherwise normal neuromorphology) in SG2 were identified already at the first episode of psychosis. SG1 had a significantly higher proportion of FEP (32%) vs. HC (19%) than SG2 (FEP, 21%; HC, 23%). Clinical multivariate signatures separated the SG1 and SG2 subgroups (balanced accuracy = 64%; *p* < 0.0001), with SG2 showing higher education but also greater positive psychosis symptoms at first presentation, and an association with symptom remission at 1-year, 5-year, and when timepoints were combined. Neuromorphological subtypes of schizophrenia are already evident at illness onset, separated by distinct clinical presentations, and differentially associated with subsequent remission. These results suggest that the subgroups may be underlying risk phenotypes that could be targeted in future treatment trials and are critical to consider when interpreting neuroimaging literature.

## Introduction

Biological heterogeneity underlying psychosis has prevented the identification and implementation of precision medicine approaches in the clinical care of this disorder. A long history of research into the relationship between brain morphology and diagnoses [1], illness stages [2], or symptoms [3] has used top-down approaches, but the lack of clinical translation [4–6] has motivated calls for bottom-up approaches to identify neuromorphological subgroups directly from brain scans [7–12]. Brain clustering is one approach that aims to find naturally occurring subgroupings of individuals defined by similar brain patterns using brain volume [7] or cortical thickness measures [11]. Such studies can identify subgroups that explain underlying psychosis heterogeneity in the neuroimaging field and lead to more precise treatment approaches if related to clinical outcomes, such as symptom remission.

To date, studies have focused on determining neuroimaging subgroups within samples of patients with psychosis in advanced illness stages [7–11, 13], reporting two [7, 8, 11, 13, 14], three [10, 15, 16], and six [17] subgroup solutions. Such studies generally find separations between groups based largely on the severity of the brain differences in the patient population related to controls—e.g., with one subgroup demonstrating widespread reductions in volume [7] or thickness [10], and the other showing fewer or no reductions [7]. Evidence also suggests that some of the elements crucial to the subgrouping may not be specific to psychosis, as similar elements are found across diagnoses [11], in depression risk samples [18], and to some degree in samples of healthy controls [10, 14, 17]. These findings point to the presence of a brain-risk phenotype that is normatively present in the healthy population but is enriched in samples of individuals with psychosis. However, a major limitation in this research has been the limited internal or external validation of the subgroups in independent samples. Furthermore, commonly used clustering algorithms used for the subgroupings are often confounded by disease-unrelated factors that influence brain variability across individuals, such as age and sex [19].

The recent PHENOM consortium aimed to address these limitations by applying a semi-supervised machine learning technique (heterogeneity through discriminative analysis; HYDRA [19]) to cluster brain MRI volume in a large sample of schizophrenia patients with internal validation (i.e., cross, split-half, and leave-site-out validation) [7]. In this work, we found a stable and highly reproducible two-subgroup solution. One subgroup of patients (SG1) demonstrated widespread smaller cortical volume [7], as also found in previous work [8, 13]. For the first time, we also found a subgroup consisting of approximately one-third of patients that showed no cortical reductions compared to healthy controls but presented with larger striatal volumes (SG2) [7]. While these morphological alterations were not associated with baseline symptoms, individuals in SG2 exhibited higher educational attainment.

In our recent follow-up work, the PHENOM subgroups were also found to be expressed in population samples both in young adults (16–23 yrs), where greater SG1 membership was found in youth with psychosis spectrum symptoms, and in adults (44–50 years) where SG1 was associated with lower cognitive performance and higher genetic risk for schizophrenia [20]. These findings suggest that SG1 is associated with normative psychosis risk that is enriched in clinical samples while SG2 is not and may even be protective [21]. A limitation of ours and other work thus far, however, is that we do not know how these subgroups are expressed at the time of a first-episode of psychosis (FEP) when there is a confirmed psychosis diagnosis but less influence of illness or treatment confounds. Determination of FEP subgroup membership is also critical because this is a clinical window when decisions may influence the symptom and functioning illness course (i.e., prognoses) [22].

Longitudinal studies examining outcomes in first-episode populations have found substantial heterogeneity, especially in trans-diagnostic samples consisting of primary diagnoses of schizophrenia, bipolar disorder, and depression with psychotic symptoms [23, 24], with consistent reports of a subgroup of individuals (~30%) with unremitting symptoms [25] in addition to a diversity of other episodic courses [24, 25]. Such unremitting courses have been previously associated with male gender, lower educational achievement, and schizophrenia or non-affective diagnoses [22], together with widespread reductions in brain volume, gyrification abnormalities [23, 25–27], and less efficient connectivity [28, 29]. In contrast, studies have found that patients who have experienced periods of remission also have larger striatal volumes, similar to what we have seen in SG2, and particularly in female individuals [28, 29].

Such top-down clinical findings suggest that our SG1 (‘lower volume’) subgroup would be less likely to achieve remission when compared to the SG2 (‘increased striatum’) subgroup, potentially via relationships with female gender, higher educational achievement, and a diagnosis of psychosis different from schizophrenia. Supporting the first hypothesis, our previous cross-sectional study found an association with illness duration in the SG1 subgroup [7] and other preliminary research found an association between relapses and a cortical thinning subgroup [10]. However, a clear limitation of existing studies is that they mostly used a cross-sectional design with a mix of patients with first-episode and chronic psychosis [8, 10]. Research is thus required that specifically investigates remission longitudinally, beginning from the very first episode of illness.

Here, we applied the pre-trained subgroup models from chronic schizophrenia to an international multi-site sample consisting of individuals with first-episode psychosis (FEP) and healthy controls. First, we determined the proportion of FEP individuals (relative to HC) that belonged to the SG1 and SG2 subgroups with the expectation that the proportion of SG1 would be higher in FEP. Second, we explored baseline clinical signatures of the subgroups with the hypothesis that SG2 would demonstrate higher educational attainment. Third, we investigated remission over 1-, 3-, and 5-year longitudinal periods with the hypothesis that subjects in SG2 would be more likely to experience illness remission.

## Methods

### Participants

We previously [7] created a two-group structural MRI statistical model in a sample with established schizophrenia from USA (*n* = 96), Germany (*n* = 145), and China (*n* = 66) in addition to controls from the same sites (*n* = 364). For the current study, we included a PHENOM subsample of 572 first-episode psychosis (FEP) patients, collected from 4 sites: Sao Paulo (*n* = 128; from two independent studies), Santander (*n* = 186), London (*n* = 122), and Melbourne (*n* = 136) (Supplemental Methods). In addition, 424 healthy control (HC) participants without Axis-I diagnoses were included from the same sites (Supplemental Methods). Sample size determination was based on discriminative effect sizes from our previous publications for machine learning imaging analyses [7, 20] and an expected 10–15% difference between subgroup proportions in baseline FEP patients and longitudinal remission [7, 20, 28]. We intentionally included samples that employed diverse sample recruitment, inclusion/exclusion criteria, and imaging protocols to assess the ability of the previously identified subgroups to generalize to a wide variety of contexts (see Supplementary Materials). Of note, none of these sites were included in the original training sample used to build the subgrouping models [7]. Local ethics committees at each site approved the studies and informed consent was obtained for all participants. Images were pooled at the Center for Biomedical Image Computing and Analytics of the University of Pennsylvania, USA.

### Sociodemographic and clinical assessments

Clinical measures included a harmonized set of basic demographic and clinical legacy data from each site collected as part of the PHENOM consortium. Demographic measures included age, sex, and proxies for functioning including: relationship status (single or not), employment status (no/yes), and highest education (1 = 12 yrs; 2 = 16 yrs; 3 = 18 yrs; 4 = tertiary education). Clinical measures included diagnoses defined by the Diagnostic and Statistical Manual of Mental Disorders (DSM-III-R, Melbourne; DSM-IV, Spain, Sao Paulo, UK) [30]. Age at illness onset was also acquired, in addition to duration of illness, duration of untreated psychosis, chlorpromazine equivalent dose (CPZ), and Global Assessment of Functioning (GAF) [31]. Symptoms were assessed with the Positive and Negative Symptom Scale (PANSS) [32]. Missing data were noted across databases and indicated in Table 1, and Supplementary Tables S4 and S10. CPZ-equivalent doses of antipsychotic medications were in different ranges for each site and the measure was z-normalized in further analyses to account for differences in calculation. Remission was defined as a binary variable (remission/no remission) according to: (1) the Andreasen symptomatic remission criteria [22] in Santander; (2) the DSM-IV course specifier (assessed with the SCID) in Sao Paulo [33]; (3) the World Health Organization Life Chart (WHO-LC) [34] in London according to symptom and functioning criteria. Follow-up timepoints were available at 1-year (Santander/Sao Paulo; *n* = 261), 3-years (Santander; *n* = 147), and 5-years (London/Sao Paulo; *n* = 135) (Supplementary Methods; Supplementary Tables S1/S2). A limited amount of data was available at 10-years from the Melbourne (*n* = 56) site with remission measured with the WHO-LC and these were not used in further analyses.

**Table 1:** Differences in baseline variables across brain subgroups in patients.

	*N* (data available)	None	SG1	SG2	Mixed	F/Chi2/KW	*p* value	Eta2/Phi
N (% of total FEP)	572	217 (38)	184 (32)	118 (21)	53 (9)			
Sites						30.25 (16)	3.97e−04	0.23
London (*n*; %)		55 (25.3)	32 (17.4)	24 (20.3)	11 (20.8)	3.87 (8)		0.08
Melbourne (*n*; %)		58 (26.7)	39 (21.2)	30 (25.4)	9 (17.0)	3.25 (8)		0.08
Santander (*n*; %)		58 (26.7)	54 (29.3)	52 (44.1)	22 (41.5)	13.28 (8)	4.06e−03	0.15
Sao Paolo (*n*; %)		46 (21.2)	59 (32.1)	12 (10.2)	11 (20.8)	20.32 (8)	1.46e−04	0.19
Age (mean;SD)	572	26.8 (7.3)	26.0 (6.6)	26.1 (7.3)	25.0 (6.1)	1.05 (3568)		0.01
Sex (male, %)	572	131 (60.4)	127 (69.0)	74 (62.7)	36 (67.9)	3.68 (8)		0.08
Marital Status (single, %)	346	75 (64.7)	84 (70.6)	58 (81.7)	34 (85.0)	9.95 (8)		0.13
Unemployed (yes; %)	281	45 (49.5)	52 (52.5)	32 (55.2)	12 (36.4)	3.34 (8)		0.08
Highest Education (1–4 scale; median)	389	3	2	3	2	40.02 (3385)	1.05E−08	0.26
Family History of Psychosis (yes, %)	449	22 (13.7)	20 (13.2)	10 (10.6)	7 (16.7)	1.01 (8)		0.04
Diagnoses								
Schizophrenia (yes; %)	555	54 (24.9)	65 (35.3)	31 (26.3)	27 (50.9)	16.51 (8)	8.92e–04	0.17
Schizoaffective (yes; %)	555	11 (5.1)	13 (7.1)	3 (2.5)	0 (0.0)	6.18 (8)		0.10
Schizophreniform (yes; %)	555	62 (28.6)	47 (25.5)	37 (31.4)	14 (26.4)	1.31 (8)		0.05
Psychosis NOS (yes; %)	555	20 (9.2)	14 (7.6)	12 (10.2)	4 (7.5)	0.75 (8)		0.04
Brief Psychotic Disorder (yes; %)	555	14 (6.5)	9 (4.9)	13 (11.0)	2 (3.8)	5.27 (8)		0.10
Drug Induced Psychosis (yes; %)	555	1 (0.5)	2 (1.1)	0 (0.0)	1 (1.9)	2.48 (8)		0.07
Bipolar-I (yes; %)	555	24 (11.1)	19 (10.3)	11 (9.3)	3 (5.7)	1.47 (8)		0.05
MDD (yes; %)	555	19 (8.8)	15 (8.2)	7 (5.9)	1 (1.9)	3.48 (8)		0.08
Onset Age (yrs; mean (SD))	431	26.1 (7.5)	24.9 (6.6)	25.5 (7.6)	23.8 (5.9)	1.38 (3427)		0.01
Duration of Illness (years; mean (SD))	421	0.7 (1.1)	0.7 (1.1)	0.7 (1.1)	1.3 (1.7)	2.62 (3417)		0.02
Duration of Untreated Psychosis (mths; mean (SD))	362	11.3 (22.7)	14.4 (28.2)	9.3 (17.3)	20.1 (41.3)	1.72 (3358)		0.01
GAF (mean (SD))	192	50.3 (31.7)	51.7 (33.3)	50.9 (35.4)	48.9 (29.9)	0.04 (3188)		0.00
PANSS Positive (mean (SD))	350	16.8 (6.9)	15.8 (7.8)	19.1 (8.1)	16.9 (9.3)	2.59 (3346)		0.02
PANSS Negative (mean (SD))	350	15.5 (7.1)	15.6 (7.5)	16.0 (6.9)	17.7 (7.8)	0.78 (3346)		0.01
PANSS General (mean (SD))	350	32.8 (10.5)	31.5 (11.5)	34.1 (9.9)	34.0 (12.2)	1.00 (3346)		0.01
PANSS Total (mean (SD))	350	65.3 (20.7)	62.9 (23.3)	69.2 (21.2)	68.3 (25.6)	1.28 (3346)		0.01
Treatments and Drug Use								
CPZ Z-norm (Mean (SD))	409	−0.1 (0.8)	0.1 (0.8)	0.2 (1.6)	0.4 (1.1)	2.88 (3405)	–	–
Treatment Duration (mths; mean (SD))	230	3.0 (2.5)	3.3 (2.6)	2.9 (2.2)	4.1 (4.8)	1.07 (3226)	–	–
Lithium (yes, %)	424	11 (7.2)	6 (4.3)	8 (8.9)	2 (4.8)	2.35 (8)	–	–
Other mood stabilizers (yes, %)	182	1 (1.8)	0 (0.0)	0 (0.0)	0 (0.0)	2.26 (8)	–	–
Antidepressants (yes, %)	284	7 (7.6)	9 (9.0)	3 (5.0)	5 (15.6)	3.18 (8)	–	–
Antiepileptics (yes, %)	102	1 (2.8)	6 (12.8)	0 (0.0)	2 (18.2)	4.52 (8)	–	–
Antipsychotic class typical/atypical (typical, %)	335	13 (11.2)	23 (21.7)	2 (2.7)	6 (15.4)	14.42 (8)	2.38e−03	0.16
Marijuana (yes, %)	314	32 (30.8)	39 (34.5)	29 (45.3)	14 (42.4)	4.31 (8)	–	–
Other illicit (yes, %)	329	37 (33.9)	38 (34.2)	27 (37.5)	9 (24.3)	1.94 (8)	–	–
Tobacco (yes, %)	181	26 (46.4)	33 (64.7)	27 (51.9)	12 (54.5)	3.74 (8)	–	–

Results corrected for multiple comparisons at a false-discovery rate (FDR) <  0.05. ANOVA used for continuous variables, Chi^2^ for binary, and Kruskall–Wallis for education variable. *PANSS* positive and negative syndrome scale, *GAF* global assessment of functioning, *CPZ* chlorpromazine equivalent dose, *MDD* major depressive disorder, *Psychosis NOS* Psychosis not otherwise specified.

### Image preprocessing

The imaging data were preprocessed using the same pipeline as in the original analyses of T_1_ images in our previous publication [7]. This included a quality control routine, followed by the application of multi-atlas segmentation (MUSE) [35] used to obtain gray and white matter regions of interest (ROI) and cerebrospinal fluid (CSF) (Supplementary Material). MUSE utilizes an ensemble of atlases coming from different scanners, field strengths, and acquisition protocols, which renders the method robust to such confounds compared to other methods [36]. Voxel-wise volumetric maps [37] were also generated, converted to the MNI space for all sites/participants, and compared between subgroups for gray and white matter. Age, sex, and site effects were corrected using a linear regression with an age-matched HC reference sample following our previous work [7, 20](Supplementary Materials). The correction of site effects was verified in the full sample and healthy controls by comparing the mean uncorrected and corrected volumes. The correction procedure was then separately applied to the voxel-wise volumetric maps.

### Application of HYDRA models

**Fig. 1:**
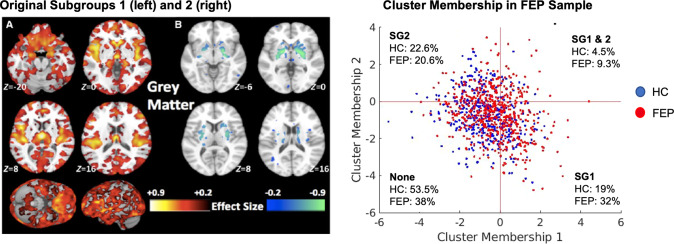
Model application to the FEP and HC samples. **A** Original subgroups in the sample of individuals with chronic schizophrenia demonstrating subgroup 1 with widespread volume reductions compared to healthy controls and subgroup 2 with no volumetric reductions and increased striatum. **B** Application of the models to the FEP sample and healthy controls defined the subgroup membership within four quadrants. A proportional difference was found across subgroups, indicating a higher proportion of FEP cases in subgroup 1 and a lower proportion within the ‘None’ category.

HYDRA [19] is a semi-supervised method that employs a supervised machine learning algorithm (support vector machine; SVM) to determine boundaries that separate controls from patients while simultaneously identifying patient-specific subgroups. Reproducibility of the subgroup solution is assessed by using an internal cross-validation cycle to identify subgroups. This technique was previously applied to volumetric ROIs of the sample with chronic schizophrenia in our original analysis and the solution was validated using split-half and leave-site-out analyses [7]. External application was conducted by applying the trained SVM models separating chronic schizophrenia individuals from controls as reported in our previous work [7] to the site-, age- and sex-adjusted ROIs of the sample used in this study containing both FEP and healthy control samples (see Supplement for details). Because SVM is a margin-based hyperplane method, each subject receives a subgroup membership score quantifying the degree to which their multivariate ROI pattern matches the subgroup label based on the distance from the hyperplanes. The continuous decision scores from the two SVMs representing each original subgroup were used in further analyses to provide binary labels within a two dimensional coordinate space (Fig. 1), such that: (1) a SG1 ‘lower brain volume’ label was given if the SG1 decision score was positive and the SG2 score was negative; (2) an SG2 ‘higher striatum volume’ subgroup label was given if the SG2 decision score was positive and the SG1 decision score was negative; (3) a SG1 + SG2 ‘mixed’ label was given if the decision score was positive for both SG1 and SG2; (4) a ‘None’ label was given if the decision score was negative for both SG1 and SG2 (see Fig. 1).

### Voxel-wise analysis

Once subgroups were defined in the FEP and HC samples, descriptive whole-brain voxel-wise volumetric analyses were conducted to provide more fine-grained brain volume differences between FEP subgroups, and separately, between the HC subgroups. In each comparison, we contrasted the ‘None’ subgroup with SG1 and SG2. Regionally linear multivariate discriminative statistical mapping (MIDAS) [38] was used for this purpose due to its demonstrated ability to detect sensitive and specific subgroup differences compared to other multivariate methods (see Supplementary Material). Because the subgroups were defined by brain differences and thus introduce circularity, results were presented descriptively to illustrate similarity with patterns reported in our previous work [7].

### Baseline and longitudinal univariate tests of subtype differences

Baseline differences in demographic characteristics, education, symptoms, and level of functioning variables were assessed across all 4 subgroups using the *F*-test (continuous) and Chi-square/Kruskall–Wallis (categorical) analyses. Remission was analyzed comparing subgroups for each timepoint using chi-squared analyses. In addition, a composite measure quantifying whether each individual available had ever remitted during any follow-up period (i.e., to identify individuals who exhibit ongoing long-term clinical impairment) was created. Longitudinal symptom data, as measured by the PANSS and the GAF-Symptoms, were also available for a subset of the sites and were analyzed with ANOVA. Two-sided tests were used for all analyses.

### Supervised machine learning analyses separating subgroups and prediction of clinical outcomes

In order to investigate baseline clinical signatures separating the four subgroup classes (SG1, SG2, SG1 + SG2 and None) a multi-group machine learning analysis was conducted employing a nested cross-validation design (25-fold outer loop; fivefold inner loop with 4 shuffled permutations) including all clinical variables outlined in Table 1. Within each training fold, the clinical data were scaled and imputed using a k-nearest neighbor approach (7 neighbors). The preprocessed training data were then forwarded to an L1-regularized SVM classifier (LIBLINEAR; *C* = $\left\{ {2^\gamma |\gamma \in \{ - 6, - 4,...4\} } \right\}$) to determine separation boundaries for each pair-wise brain subgroup. These models were applied without modification to the inner loop test data for all hyperparameters. Optimized models were applied without modification to the outer-loop held-out test data to obtain final accuracies. Permutation testing was conducted (10,000 iterations; labels swapped) to obtain significance levels for balanced accuracy estimates. The cross-validation ratio [39] and sign-based consistency [40] of feature weight measures were used to determine variable importance and significance. The same clinical variables and parameters within the multigroup setting were also employed to predict remission at each 1-, 3-, and 5-year timepoints and to determine any incidence of remission across all follow-up periods (i.e., nested cross-validation, preprocessing, and L1-regularized SVM). Based on our hypotheses regarding remission, the binary labels representing SG1, SG2, or SG1 + SG2 subgroup membership were added to the clinical measures in addition to the continuous SG1 and SG2 membership scores (see Fig. 1; we included both binary and continuous scores in the context of L1-regularized variable selection to determine which was maximally predictive).

## Results

### Baseline and follow-up sample characteristics

Compared to controls, FEP cases had higher rates of single marital status and unemployment, but did not differ in age, sex, or education (Table S3). Substantial site differences were found across most demographic and clinical measures, except sex, the presence of a diagnosis of psychosis not otherwise specified (NOS), and duration of untreated psychosis (Table S4) (Supplemental Methods). For example, Melbourne included patients who were 5-years younger and had higher psychosis symptom severity (PANSS Total) compared to other sites, while Sao Paulo contained more cases with a diagnosis of schizophrenia (PSYCLASS sample) and lower educational attainment (PSYCLASS/ESNA samples). Follow-up participants were significantly older and had a higher prevalence of schizophrenia diagnoses, but less symptom load overall, and more use of illicit substances (Table S5).

### Validation of neuromorphological subgroups at the time of the first psychotic episode

Site effects were effectively corrected (Figs. S1 & S2). Following application of our previously trained HYDRA model [7] we obtained the following subgroup split: FEP (*n* = 572): SG1, *n* = 184(32%); SG2, *n* = 118(21%); SG1 + SG2, *n* = 53(9%); None, *n* = 217(34%); HC (*n* = 424): SG1, *n* = 82(19%); SG2, *n* = 96(23%); SG1 + SG2, *n* = 19(5%); None, *n* = 227(54%) (Fig. 1, Table 1). The higher proportion of FEP in SG1 compared to the other subgroups was significant (*X*^2^(8) = 36.5, *p* = 5.96e−08). These findings thus confirm that the two neuromorphological subtypes we had identified in patients in the advanced illness stages are already present at illness onset.

**Fig. 2:**
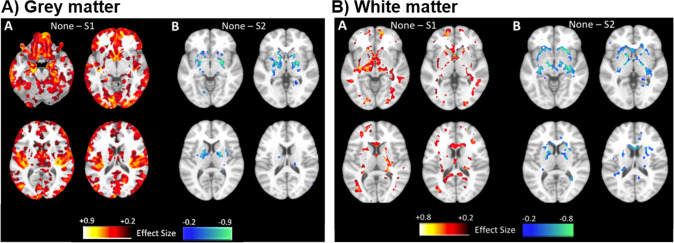
Subgroup comparisons in the FEP sample. Gray matter (**A**) and white matter (**B**) comparisons of subgroup SG1 (left) and SG2 (right) when compared to cases in the ‘None’ subgroup in the FEP sample. **A** Decreased gray matter volume was found in the FEP subgroup relative to the ‘None’ classification, including the caudate, whereas relatively increased gray matter was found in SG2 subcortical areas including the striatum. **B** Decreased white matter was also found in the SG1 subgroup in areas including the internal capsule.

When whole-brain voxel-wise maps of the SG1 subgroup were compared to those of the ‘None’ subgroup in FEP, the SG1 subgroup showed smaller widespread cortical volumes coupled with smaller volume in some parts of caudate (Fig. 2). In contrast, larger gray matter volumes in SG2 subgroup were restricted to subcortical structures including the striatum. White matter was also smaller in SG1, mainly in subcortical regions adjoining the striatum, whereas white matter was larger in SG2 (Fig. 2). Because healthy control participants were also included in the SG1 and SG2 subgroups, we tested whether these subgroups demonstrated similar patterns to the patients and our previous work in population samples. Voxel-wise analyses were thus repeated in healthy controls comparing each subgroup definition to the ‘None’ subgroup to visualize the separation indicated by the application of the subgroup models (Figs. S3 and S4). These analyses demonstrated an SG1 pattern that was restricted in spatial extent and magnitude when compared to that of the full sample, although larger striatal volumes were also found in the HC of in SG2 (Fig. S3) and the pattern was no different from that of FEP patients (Fig. S4). Results were maintained when controlling for antipsychotic dose and type (i.e., atypical vs. typical; Supplementary Materials ‘Investigation of Medication Effects’; Fig. S5) and when investigating volume differences within individual sites (Fig. S6). Supplementary analyses in a small sample of SG1 + SG2 individuals showed both decreased cortical volume and increased striatal volume (Fig. S7). Global and distributed volume reductions in SG1 were maintained when controlling for intracranial volume (Fig. S8).

### Differences in clinical baseline characteristics between SG1 and SG2 subgroups in FEP and HC

Subgroups were not different in age or sex, but a higher proportion of SG1 cases came from the Sao Paulo sample (i.e., containing more individuals with a schizophrenia diagnoses and lower education) and a higher proportion of SG2 cases came from the Santander site (Table 1). Importantly, these results occurred in the context of our site correction. The SG1 and SG1 + SG2 subgroups also included more individuals with a diagnosis of schizophrenia (SG1, 35%; SG1 + SG2, 50.9%). Individuals in SG1 (both FEP and HC) were also more likely to have a lower educational attainment at uncorrected levels (Table S6). SG1 were also prescribed proportionately more typical antipsychotics (Table 1).

### Prediction of subgroup membership from clinical signatures

**Fig. 3:**
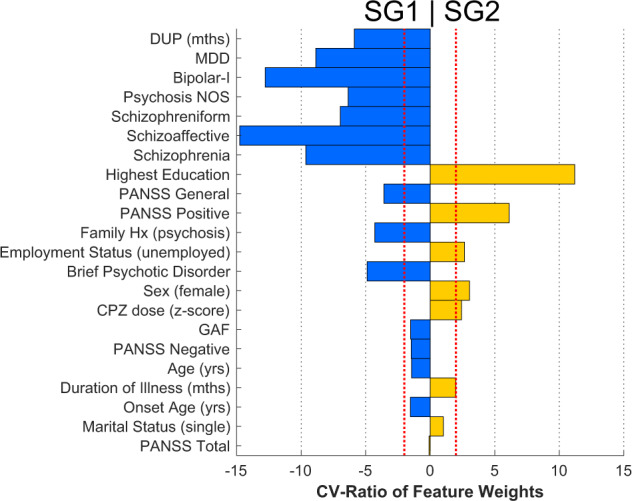
Baseline multivariate signatures related to the classification of subgroups. Cross-validation ratio is plotted (*x*-axis) in and sign-based consistency significance is indicated by colored bars (yellow, positive weights; blue, negative weights). SG2 was separated from the SG1 subgroup by positive weights (commonly indicating relative increases) in highest education, positive symptoms, unemployment, female sex, and CPZ dose (*z*-scored within each site). Prediction of SG2 was also associated with negative weights (commonly indicating relative decreases) of duration of untreated psychosis (DUP), psychotic diagnoses, general psychosis symptoms, and family history of psychosis. PANSS positive and negative syndrome scale, GAF global assessment of functioning, CPZ chlorpromazine equivalent dose, MDD major depressive disorder, Psychosis NOS Psychosis not otherwise specified.

In patients, multigroup machine learning analyses revealed that the highest accuracy of subgroup separation based on clinical variables (see below) was associated with the SG1 vs. SG2 comparison (Table 2). SG2 membership was separated from SG1 with a sensitivity of 66% (balanced accuracy (BAC) = 64.03%) by a pattern including: education, higher positive symptoms, unemployment, female sex, and higher CPZ dose (z-scored within each site to account for site differences) (Fig. 3). Longer duration of untreated psychosis, diagnoses (including comorbidity), general psychosis symptoms, and family history of psychosis significantly predicted SG1 membership. Other subgroup comparisons were non-significant (although a trend was noted for SG1 vs. SG1 + SG2; Table 2).

**Table 2:** Multigroup prediction of brain subgroup membership at baseline using sociodemographic and clinical variables.

	TP	TN	FP	FN	Sens (%)	Spec (%)	BAC (%)	NPV (%)	AUC	BAC *p* value 10,000 perms
SG2 vs. SG1	78	114	70	40	66.10	61.96	64.03	74.03	0.67	0.0001
SG2 vs. None	71	44	138	30	70.30	24.18	47.24	59.46	0.45	0.84
SG2 vs. Mixed	86	27	25	31	73.50	51.92	62.71	46.55	0.60	0.07
SG1 vs. None	91	126	89	88	50.84	58.60	54.72	58.88	0.56	0.08
SG1 vs. Mixed	107	29	24	77	58.15	54.72	56.43	27.36	0.61	0.06
None vs. Mixed	149	28	25	61	70.95	52.83	61.89	31.46	0.62	0.13

*TP* true positive, *TN* true negative, *FP* false positive, *FN* false negative, *sens* sensitivity, *spec* specificity, *BAC* balanced accuracy, *PPV* positive predictive value, *NPV* negative predictive value, *AUC* area under the curve.

### Illness remission across subgroups

Individuals in SG2 were more likely to have experienced at least one period of symptom remission across all follow-up timepoints (SG1, 57%; SG2, 78%; *X*^2^(8) = 9.8, *p* = 0.02; Table S7). For individual follow-up points, higher 1-year remission in SG2 was found at uncorrected levels (SG1, 52.7%; SG2, 73.2%; *X*^2^(8) = 7.7, *p* = 0.05; Table S7). To provide a complementary perspective on the binary subgroup labels we also examined the relationship between membership strength (as the continuous decision scores) and likelihood of remission. We found that SG2 membership scores were significantly higher in cases who demonstrated at least one period of remission across all timepoints (*t*(352) = −2.97, *p* = 0.003), while there were no differences for SG1.

### Prediction of remission from clinical variables and subgroup membership

**Fig. 4:**
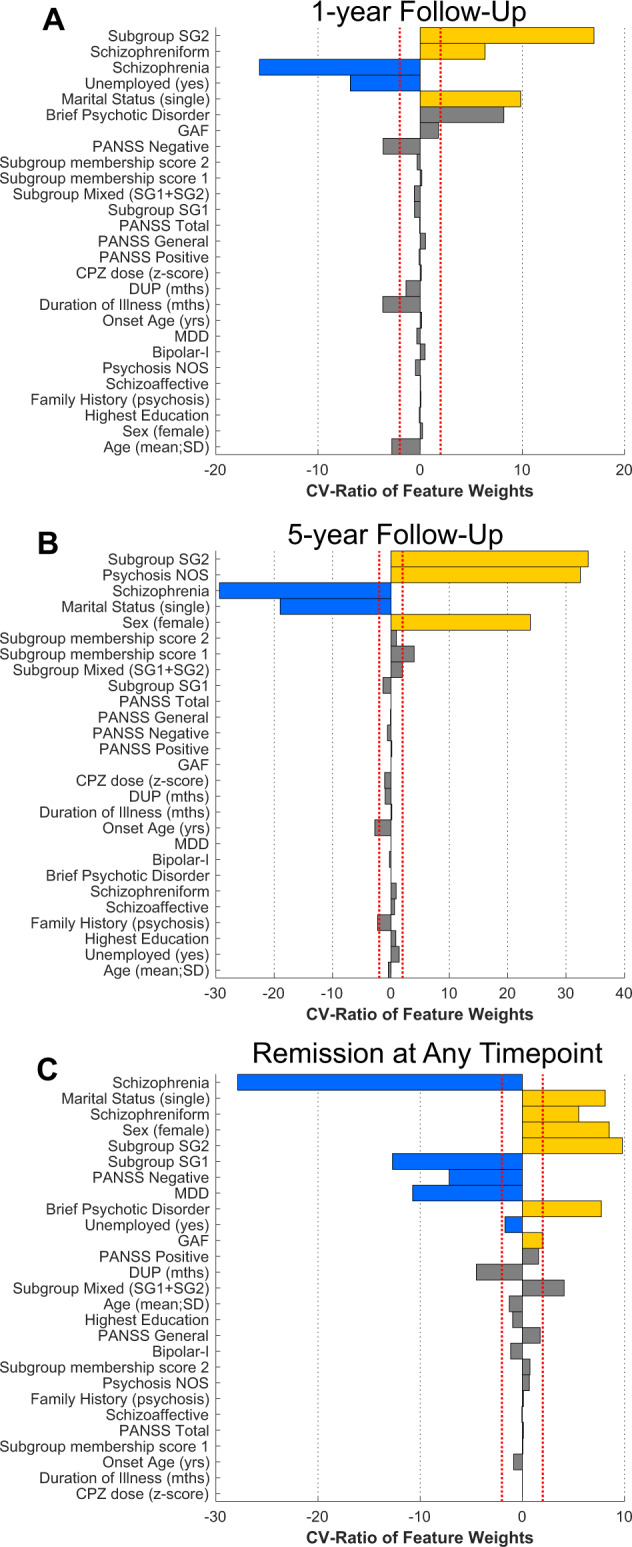
Baseline prediction of remission outcomes at 1-year (**A**), 5-year (**B**), and at any point across all follow-up periods (**C**). Cross-validation ratio (CV-Ratio) of feature weights indicating most consistently selected variables is presented (*x*-axis) and colored bars indicate those that are significant using the sign-based consistency measure. **A** At 1-year (data available from Santander and Sao Paulo; Table S1), the SG2 membership positively predicted remission in combination with increased diagnoses of schizophreniform disorder, increased single marital status, and less schizophrenia diagnoses and unemployment. **B** At 5-years (London/Sao Paulo; Table S1), SG2 significantly predicted remission together with a psychosis “not otherwise specified” diagnosis, female sex, less schizophrenia diagnoses, and less single marital status. **C** Prediction of remission included increased single marital status, schizophreniform or brief psychotic disorder diagnoses, female sex, and global assessment of functioning (GAF) in addition to less schizophrenia diagnoses, negative symptoms, and major depressive disorder. Increased SG2 was associated with remission, while decreased SG1 membership (relative to the other variables in a multivariate analysis), was associated with remission. DUP duration of untreated psychosis, PANSS positive and negative syndrome scale, GAF global assessment of functioning, CPZ chlorpromazine equivalent dose, MDD major depressive disorder, Psychosis NOS Psychosis not otherwise specified.

Supervised machine learning was used to predict remission across timepoints using the clinical variables in addition to the SG1, SG2, and SG1 + SG2 labels to determine if subgroup membership was associated with more positive outcomes. The ‘None’ subgroup label was not included as it was not included as a hypothesis and we did not find sufficient separation at baseline. At the 1-year follow-up point (Santander/Sao Paulo), the balanced accuracy (BAC) was 64.2% (sensitivity, 60%; specificity, 68.5%; Table 3). The pattern predicting higher likelihood of remission included membership in SG2, increased schizophreniform disorder diagnoses, increased single marital status, and reduced schizophrenia diagnoses and unemployment (Fig. 4). While subgroup membership was not among baseline variables that predicted remission at 3-years (Santander), membership of SG2 predicted remission status at 5-years (London/Sao Paolo; BAC = 59.4%; sensitivity, 51.6%; specificity, 67.2%), together with a diagnosis of psychosis NOS, and female sex, and reduced likelihood of a schizophrenia diagnosis and single marital status. At 5-years (London/Sao Paulo; BAC = 59.4%; sensitivity, 51.6%; specificity, 67.2%) the pattern included higher SG2 membership, increased psychosis NOS, and female sex in addition to less schizophrenia diagnoses and single marital status.

**Table 3:** Analyses predicting remission across timepoints using sociodemographic, clinical, and brain subgroup membership variables.

Analysis	N	TP	TN	FP	FN	Sens (%)	Spec (%)	BAC (%)	PPV (%)	NPV (%)	AUC	BAC *p* value 10,000 perms
1-year	261	90	76	35	60	60	68.46	64.23	72	55.88	0.69	0.03
3-years	135	26	60	31	18	59.09	65.93	62.51	45.61	76.92	0.60	0.03
5-years	126	32	43	21	30	51.61	67.19	59.40	60.38	58.90	0.61	0.05

*TP* true positive, *TN* true negative, *FP* false positive, *FN* false negative, *sens* sensitivity, *spec* specificity, *BAC* balanced accuracy, *PPV* positive predictive value, *NPV* negative predictive value, *AUC* area under the curve.

### Supplementary analyses of combined timepoints, site, antipsychotics, schizophrenia diagnosis, drug use, and clustering algorithms

Combining remission information across timepoints increases sample size for machine learning predictions. Remission prediction at any time across 1-, 3-, and 5-year periods was predicted by a pattern that included increased likelihood of SG2 membership and decreased likelihood of SG1 membership at uncorrected levels, using the sign-based consistency measure of variable significance (Fig. S9).

Site differences were controlled for in brain volume measures (Figs. S1 and S2) prior to obtaining the subgroup labels. However, clinical analyses were conducted without site control because of clinical differences in inclusion/exclusion criteria, study populations, and experimental designs (Supplementary Materials), which could meaningfully influence membership into subgroups and the clinical prediction of remission. Controlling clinical analyses for site in this case therefore can control for important clinical variance related to outcomes. However, we repeated the analyses while controlling for site to determine if relationships with brain subgroups were maintained. Even when we controlled for site, SG2 membership remained predictive of remission at across all timepoints (Fig. S9) and at 5-years (Fig. S10), in addition to 1-year at uncorrected levels (Fig. S10). We also controlled for antipsychotic effects (dose and type) and the main findings were largely unchanged (Supplementary Materials ‘Investigation of Medication Effects’; Figs. S11 and S12).

In our analyses, a diagnosis of schizophrenia was a consistent feature associated with lower likelihood of remission and this raised the possibility that SG2 membership was mediated by sampling diagnoses differences in samples across sites. We thus controlled for schizophrenia diagnosis and repeated the analysis of remission across all timepoints controlling for schizophrenia diagnosis, to reduce the number of comparisons. Results confirmed that remission was associated with higher SG2 membership and lower SG1 membership, negative symptoms, and major depressive disorder diagnoses (Fig. S13). We also controlled for illicit drug use in clinical remission analyses and found similar results (Fig. S13). Finally, we compared the clustering results from the semi-supervised technique (HYDRA) with those form a completely unsupervised technique (k-means + +). Results demonstrated broadly similar brain volumetric results, evidencing considerable stability, except relationships between SG2, education, and remission were not found (Supplementary Materials ‘Investigation of subgroup separation with unsupervised methods’; Fig. S15; Tables S11–S15).

## Discussion

In this study, we used a large international multi-site heterogeneous sample of individuals with a first-episode of psychosis to validate the presence of the two data-driven neuromorphological subgroups originally derived from a sample of individuals with chronic schizophrenia. We also evaluated whether these subgroups were related to remission over the subsequent course of illness. Our findings show for the first time that these two subgroups [7], SG1 with lower widespread cortical volumes and SG2 with larger striatal volume but otherwise normal brain morphology, are already apparent at the first presentation of illness. Furthermore, our data show a distinct clinical signature separating these subgroups, and that the subgroup presenting with only increased striatal and pallidum volume (SG2) was significantly more likely to achieve remission in subsequent years. These findings support the presence of reproducible neuromorphological subgroups in individuals with psychosis that may help delineate the heterogeneity of brain structure reported in previous neuroimaging research [1, 41, 42]. With further follow-up studies, the findings could also be crucial in informing future research to refine stratified therapeutic approaches and outcome prediction.

The higher proportion of patients with psychosis within the SG1 subgroup relative to HC agrees with our hypothesis based on our previous research in population samples [20]. The findings suggest that the subgroup solutions we identified are not the result of a long duration of illness [7, 8, 10, 11, 13, 14, 16, 17], but are already evident at illness onset, across a wide range of psychosis diagnoses, and before any potential effect of long-term pharmacological treatment. Notably, a similar subgroup to SG1 (but not SG2) has recently also been found in previous unmedicated first-episode schizophrenia samples [16]. In the context of our previous reports of a relationship between SG1, schizophrenia genetic risk, and subthreshold psychosis symptoms [20], the results further point to the presence of a biological vulnerability that may specifically increase risk for the expression of illness in some individuals (i.e., 32% of the FEP sample in this study). Such results support our previous neurodevelopmental hypothesis of SG1 by providing evidence for a potential brain-diathesis model, whereby an existing vulnerability could combine with other risk factors in order to trigger the illness; e.g., family history as found in our multivariable model when compared to SG2 [43] (for further discussion of neurobiological hypotheses see Supplementary Materials). This neurodevelopmental hypothesis was supported by supplementary analyses showing that decreased total brain volume and voxel-wise decreases remain after correction for intracranial volume. The results also contextualize existing neuroimaging research [41] by suggesting that smaller brain volumes are only evident for some individuals with a first episode psychosis.

The equal proportions of FEP patients and HC in SG2 additionally supports our previous population-based research showing the same lack of enrichment [20]. These results suggest that SG2 membership is more likely to be normatively present and does not increase FEP risk. In comparison to SG1 membership, we characterized the sociodemographic and clinical signature to find that SG2 membership was associated with higher education in addition to higher rates of positive symptoms, unemployment, female sex, and chlorpromazine equivalent dose. These results are important as they suggest that, for some individuals in specific brain-based subgroups, the presence of positive symptoms is not necessarily associated with smaller brain volume when examined in the context of the multivariable signature (for example, when higher education and female sex are also considered). Such findings contextualize previous reports of the association between positive symptoms and smaller brain volumes [3] by suggesting that this does not hold for some individuals. It is notable that such a finding has been hypothesized in historical theories of a ‘Type-I’ (non-deficit) schizophrenia suggesting the presence of positive symptoms with less structural brain changes compared to a ‘Type-II’ presentation associated with impairment and negative symptoms [44]. In our previous work defining the subgroups [7] we hypothesized that the SG2 subgroup was related to hyperdopaminergic mechanisms, which is now further contextualized by our current results showing a relationship between SG2, female sex, and remission, due to evidence of: increased striatal volumes in remitting females [28, 29], sex differences in striatal dopamine synaptic concentrations and receptors [45], and sex differences in response to antipsychotics [46] (see Supplementary Materials for details).

In univariate tests, remission rates were higher in the SG2 subgroup when timepoints were combined and at 1-year at uncorrected levels. Multivariable remission signatures at 1-year, 5-year, and combined timepoints included SG2 membership combined with decreased schizophrenia diagnosis (and increased diagnoses of schizophreniform, psychosis not-otherwise-specified, and brief psychotic disorder). Other notable variables included in the models with SG2 membership were: reduced unemployment (1-year), female sex (5-years), and decreased SG1 membership (combined timepoints). These results support our hypothesis of increased chances of remission in SG2 derived from top-down studies [28, 29], but add to this research by suggesting that this relationship may be mediated by a premorbid brain phenotype. The findings also highlight how the brain subgroup membership combines with known clinical signatures associated with remission, such as female gender, higher education, and a diagnosis other than schizophrenia [22–25, 47–49]. When combined with other variables across timepoints, the negative relationship between SG1 membership and remission also agreed with previous research [23, 25–27].

Against the background of the SG1 and SG2 subgroups we identified, it is important to highlight that approximately 34% of FEP individuals did not show either brain signature and were considered to have no subgroup membership (i.e., ‘None’). Multivariable signatures separating this subgroup from the others were also non-significant, potentially supporting the presence of clinical and neuromorphological heterogeneity in presentations. Given that these individuals were also experiencing a psychosis, further research should further investigate this subgroup to potentially detect additional subgroup solutions and finer-grained differences (e.g., using functional MRI or diffusion tensor imaging) that may reveal more precise relationships with symptoms and outcomes in these individuals. In addition, further research in larger samples could consider the SG1 + SG2 subgroup due to its mixed brain signature and higher proportion of individuals with a diagnosis of schizophrenia. However, overall the SG1, SG2, SG1 + SG2, and ‘None’ results highlight how such MRI heterogeneity may influence MRI findings [1, 41, 42] and ultimately obscure biomarker identification in early stage psychosis neuroimaging research [4–6]. Our study thus emphasizes the need for more research on biologically-based individual differences that also accounts for the potential presence of premorbid and normatively present brain diversity [7, 8, 10, 11, 13, 14, 17].

## Strengths and limitations

This study has multiple strengths as it provides the first replication of the presence of neuromorphological subgroups from a chronic schizophrenia cohort in a large multisite sample of patients at illness onset—a group less confounded by the effects of chronic illness (and long-term treatment)—and in healthy controls. As such, we replicated findings across diverse study protocols and demonstrated the crucial associations between these brain subtypes and clinical variables, suggesting a high degree of generalizability of these subgroups. Furthermore, we also examined the relationship between subgroups across different psychosis diagnoses and with longitudinal outcome data on up to 5-years after the baseline assessment.

However, there are also some important limitations to consider. First, while we believe this has been a strength in generalizability, we should consider the potential effect of differences in MRI acquisition and subject recruitment protocols across sites. We implemented site control procedures that mitigated MRI site variance and controlled for site in clinical analyses, although remaining effects are possible. A related point is that missing variables within each site were addressed using imputation within cross-validation routines during clinical analyses. Nevertheless, these site effects were partly controlled in supplementary analyses that reinforced our finding of a relationship between remission and membership of the SG2 subgroup. Second, although we did not find antipsychotic effects on brain volume or clinical outcomes at baseline, longitudinal treatment could influence remission signatures and needs to be investigated in future research. Third, the baseline and follow-up predictive accuracy related to the clinical associations (e.g., with SG2) was modest (60–69%). Fourth, recreational drug use could be longitudinally investigated to determine interactions with brain signatures and remission. Fifth, the interaction with cognition needs to be considered given the potential relationships with subgroups [20] and remission [50]. Further studies are needed, especially in consortium large samples consisting of clinical high-risk for psychosis groups with homogeneous clinical and cognitive tests, to further clarify these aspects (e.g., PRONIA [39]). Studies in controlled treatment trials could also further investigate differential treatment effects over time.

## Conclusion

This study validated the presence of specific brain subgroups, originally found in chronic schizophrenia study samples, in a clinically heterogeneous first-episode psychosis sample and demonstrated a significant relationship of these baseline clinical signatures and subsequent symptomatic remission. The results suggest the possibility of normatively present, premorbid brain types that influence first presentation and outcomes in FEP. Furthermore, they provide an initial, but important, indication that brain morphology could help to inform stratification approaches in the treatment of psychosis.

### Supplementary information

Supplementary Materials

## Data Availability

HYDRA (https://github.com/evarol/HYDRA) and MIDAS (https://github.com/evarol/MIDAS) code used in this study are publicly available. The machine learning analyses were conducted with NeuroMiner software available at https://github.com/neurominer-git. Additional code used to assign subgroups and compute signatures is available at https://github.com/ganchand/AJP_Codes.
