# Supplementary material for: Psychosis brain subtypes validated in first-episode cohorts and related to illness remission: results from the PHENOM consortium
Source: Mol Psychiatry. Author manuscript; Available in PMC 2023 Oct 23. (PMC10575777; doi:10.1038/s41380-023-02069-0)
Supplement: supplementary [file NIHMS1910466-supplement-supplementary.docx]

Psychosis brain subtypes validated in first-episode cohorts and related to illness remission: results from the PHENOM consortium

Dominic B. Dwyer,^4,22,23#^ Ganesh B. Chand,^1,2,#^ and the PHENOM Consortium

**Supplementary Materials**

**Methods and Results**

[Supplementary Methods 4](#_Toc127794283)

[1. Participants 4](#_Toc127794284)

[2. Image Acquisition 6](#_Toc127794294)

[3. Preprocessing and MUSE segmentation 8](#_Toc127794305)

[4. Inter-site image harmonization and correction of covariates 8](#_Toc127794306)

[5. Model Development in Established Schizophrenia Sample and Application to FEP Sample: HYDRA 9](#_Toc127794307)

[6. Voxel-wise subgroup comparisons: MIDAS 10](#_Toc127794308)

[Supplementary Results 10](#_Toc127794309)

[1. Investigation of medication effects 10](#_Toc127794310)

[2. Investigation of differences in the follow-up population only 12](#_Toc127794311)

[3. Elaboration on the neurobiological significance of the two subtypes 13](#_Toc127794312)

[4. Investigation of subgroup separation using unsupervised methods 14](#_Toc127794313)

[**References** 42](#_Toc127794314)

**Figures**

[Figure S1. Illustrates raw data and harmonized data where our harmonization steps minimize site differences. 17](#_Toc127795175)

[Figure S2**.** Raw data and harmonized data across all sites only in the healthy control population.. 18](#_Toc127795176)

[Figure S3**.** Voxel-wise differences in healthy control subjects between the “None” brain subgroup and both SG1 and SG2 subgroups. 18](#_Toc127795177)

[Figure S4. Comparison between the healthy control and patient cohorts within each of the subgroups.. 19](#_Toc127795178)

[Figure S5. Subgroup and brain differences when controlling for antipsychotic dose (z-scored chlorpromazine equivalent) and antipsychotic type (typical versus atypical). 20](#_Toc127795179)

[Figure S6. Brain patterns found when investigating effects within sites. Widespread decreases were found in SG1 across sites and increased striatum was found in SG2. 20](#_Toc127795180)

[Figure S7. GM comparison of SG1+SG2 compared to ‘None’ in FEP patients showing widespread cortical deficits and very mild increase in striatal volume. 20](#_Toc127795181)

[Figure S8. Comparison of the brain subtypes after correction for intra-cranial volume (ICV). 21](#_Toc127795182)

[Figure S9. Prediction of remission at any timepoint with no site control (A) and with regression of site (B) 22](#_Toc127795183)

[Figure S10. Regression of site within the training samples of the cross-validation routine prior to prediction of remission resulted in signatures that retained the SG2 subgroup label at 1- and 5-years. 22](#_Toc127795184)

[Figure S11. Analysis of baseline and follow-up predictive patterns while controlling for antipsychotic dose (z-scored chlorpromazine equivalent) and type (typical versus atypical). 23](#_Toc127795185)

[Figure S12. Analysis of baseline and follow-up predictive patterns while controlling for antipsychotic dose (z-scored chlorpromazine equivalent) and type (typical versus atypical). 24](#_Toc127795186)

[Figure S13**.** Controlling for a diagnosis of schizophrenia or drug use clarified the relationship with remission in analyses of all timepoints. 25](#_Toc127795187)

[Figure S14. Baseline comparison of SG1 versus SG2 only in follow-up individuals. A similar pattern of increased diagnoses was found for SG1 (blue) and increased education in SG2 (yellow). 26](#_Toc127795188)

[Figure 15. The original subgroups defined by Chand et al. (2020) compared with those generated through k-means++ clustering. 26](#_Toc127795189)

**Tables**

[Table S1. Questionnaires used in each center to evaluate remission. 27](#_Toc127795190)

[Table S2. Number of remissions at each follow-up time period for each site (remission/total). 27](#_Toc127795191)

[Table S3. Description of case/control groups used in analyses 27](#_Toc127795192)

[Table S4. Baseline demographic, clinical and medication differences across sites in FEP. 29](#_Toc127795193)

[Table S5. Analysis of differences between baseline and follow-up samples 30](#_Toc127795194)

[Table S6. Comparison of baseline demographics across subgroups in healthy control subjects. 32](#_Toc127795195)

[Table S7. Number of cases who remitted within each subgroup with percentage compared to non-remitters within the subgroup 32](#_Toc127795196)

[Table S8. Analysis of medication associations with brain subgroups (FDR significance across 4 comparisons) 33](#_Toc127795197)

[Table S9. Comparison of subgroups only in individuals with follow-up data. 33](#_Toc127795198)

[Table S10. Missingness and test of Missing Completely At Random (MCAR) in the variables used in baseline and longitudinal analyses 35](#_Toc127795199)

[Table S11. K-means++ subgroup memberships compared to the original subgroup definitions in the discovery sample. Overlap was most prominent for SG1, but SG2 subgroup membership was mixed. 36](#_Toc127795200)

[Table 12. K-means++ subgroups in the first-episode sample: discovery sample using only patients 36](#_Toc127795201)

[Table S13. K-means++ subgroups compared across remission timepoints: discovery using only patients 38](#_Toc127795202)

[Table S14. K-means++ subgroups in the first-episode sample: discovery sample using patients and healthy controls 39](#_Toc127795203)

[Table S15. K-means++ subgroups compared across remission timepoints: discovery using patients and healthy controls 41](#_Toc127795204)

# Supplementary Methods

## Participants

Participants can be divided into the sample with established schizophrenia from the original PHENOM paper describing the creation of the clustering models representing the SG1 and SG2 subgroups^1^(defined here as ‘PHENOM Model Development’) and the FEP sample used in the current study (defined here as ‘PHENOM Model Application’).

## PHENOM Model Development

In brief, cases (age range: 16-45 years) with established diagnoses of schizophrenia (n=307) and healthy controls (n=364) were derived from existing databases centred in USA, Germany, and China. Diagnostic assessment employed the Structured Clinical Interview for DSM-IV and included a review of clinical records.

## USA (Pennsylvania)

In USA, participants were collected from the University of Pennsylvania and exclusions were: a) history of substance abuse in the past six months; b) medical or neurological disorder; c) for controls only, the presence of any DSM-IV psychiatric disorder; d) MRI contraindications.

## Germany (Munich)

In Germany, participants were recruited at Ludwig-Maximilians University and exclusion criteria were: a) other psychiatric or neurological diseases; b) past or present alcohol use disorder or consumption of illicit drugs; c) head trauma with loss of consciousness or electroconvulsive treatment; d) insufficient knowledge of German; e) IQ < 70. Healthy controls with a positive family history of mental illness (first degree relatives) were excluded.

## China (Tianjin)

In China, participants were recruited at Tianjin Medical University General Hospital. Diagnosis was assessed following the consensus of two clinical psychiatrists using the DSM-IV/SCID. Exclusion criteria were MRI contraindications, pregnancy, history of systemic medical illness, central nervous system disorder and head trauma, and substance abuse within the last three months or lifetime history of substance abuse or dependence. Healthy controls with a history of psychiatric disease or a first-degree relative with psychosis were also excluded.

## PHENOM Model Application

A PHENOM subsample of 572 first-episode psychosis (FEP) patients was used for this study with samples collected from 4 sites: Brazil (n=128), Spain (n=186), UK (n=122), and Australia (n=136). A further 424 healthy control (HC) subjects were included from the same sites. Details within each site can be found below.

## Brazil (Sao Paulo)

Cases were drawn from two studies affiliated with the Institute of Psychiatry, University of Sao Paulo: PSYCLASS and ESNA. PSYCLASS participants only were included in follow-up data.

For PSYCLASS, details are available in previous work^2, 3^. Cases were drawn from a population-based incidence and case-control study of a specific geographic region of Sao Paulo, Brazil (900,000 inhabitants total). Cases were identified by surveillance of all people between 2002 to 2005 who made contact for the first time with local health services (from a predefined geographical area) due to psychotic symptoms regardless of their severity (both out-patients and in-patients were recruited), duration of illness, or compliance to treatment. Inclusion criteria for patients at baseline were age 18-50 years and a diagnosis of psychotic disorders according to DSM-IV-TR criteria (295-298) assessed with the Structured Clinical Interview for DSM-IV (SCID)^4^. People with psychotic disorders due to a general medical condition or substance-induced psychosis were excluded. In order to obtain a population-based sample of controls, next-door neighbours were contacted and screened to exclude the presence of psychotic symptoms^5^. Additional exclusion criteria for both groups were: a) history of head injury; b) presence of neurological disorders or any organic disorders that could affect the central nervous system; and c) contraindications for MRI. Exclusion criteria specific to the control group were personal history of psychosis or other Axis-I disorders, except substance misuse or mild anxiety disorders. MRI data were included from subjects specifically with first-episode schizophrenia. A total of 35 cases were followed up after 5 years (SD=10months) and assessed with the DSM-IV course specifier (Table S1/S2).

For ESNA, details are available in previous publications^6^. FEP patients aged 16-50 years were referred to the Institute of Psychiatry, University of Sao Paulo after contact with mental health services from the metropolitan area of Sao Paulo city. At study entrance, a clinical interview and the SCID^4^ was conducted. Inclusion criteria was based on a DSM-IV criteria for any non-affective FEP for less than 6 months (i.e., schizophreniform disorder, brief psychotic disorder, delusional disorder, and psychotic disorder not otherwise specified). Exclusion criteria were psychoses related to a substance use disorder or medical condition. Patients fulfilling criteria for other DSM-IV disorder (except mild anxiety; e.g., specific phobia) were excluded. Healthy volunteers were recruited through advertisement in the local community and were free from mental disorders and had no known history of diagnosed psychotic or mood disorders in their first-degree relatives. Exclusion criteria for both groups were: a) previous intake of any psychopharmacological drugs other than benzodiazepines; b) history of substance dependence or abuse; c) presence of any medical disorder that could affect the central nervous system; d) mental disability; e) history of head trauma with loss of consciousness; and f) contraindications for MRI scanning. No subjects from ESNA were followed-up over time.

## UK (London)

Participants are described in previous papers^7^. Participants were recruited from the South London and Maudsley Foundation Trust and scanned at the Institute of Psychiatry, Psychology and Neuroscience. All patients meeting ICD-10 criteria for a diagnosis of psychosis (codes F20–F29 and F30–F33) (World Health Organization, 2004) were invited to participate in the study; patients with a diagnosis of organic psychosis were later excluded. HC were recruited through local advertisement from the same geographical areas as patients. A screening tool (Psychosis Screening Questionnaire)^5^ was used to exclude the presence of psychotic symptomatology or a history of psychotic illness in healthy controls. Additional exclusion criteria for all participants included learning disabilities (based as an IQ < 70), current or past neurological illness, brain injury with the loss of consciousness for more than 1 h and suspected or confirmed pregnancy. FEP cases were assessed with the World Health Organisation Life Chart (WHO-LC) at a 5-year follow-up period (Tables S1/S2).

## Spain (Santander)

Participants are described in previous papers^7^. Individuals with FEP were recruited from both inpatient units and community mental health care centres. Patients were included if they met the following criteria: (1) age 15–60 years; (2) DSM-IV criteria for a principal diagnosis of schizophrenia, schizophreniform disorder, schizoaffective disorder, brief reactive psychosis or not otherwise specified psychosis; and (3) no prior treatment with anti-psychotic medication or, if previously treated, a total life time of adequate anti-psychotic treatment of <6 weeks. Patients with DSM-IV diagnoses of mental retardation or substance dependence (except nicotine dependence) were excluded. Age- and gender-matched HC were recruited from the community through advertisements and were screened for current or past history of psychiatric, mental retardation, neurological or general medical illnesses, including substance dependence and significant loss of consciousness, as determined by using an abbreviated version of the Comprehensive Assessment of Symptoms and History (CASH) (Andreasen, Flaum, & Arndt, 1992). The absence of psychosis in first-degree relatives was confirmed by clinical records and family interview. FEP cases were followed-up at 1-, 3-, and 10-year intervals and assessed with the PANSS to determine the Andreasen symptomatic remission^8^(Table S1/S2).

## Australia (Melbourne)

Participant details are included in previous papers^9^. Participants were recruited from the Early Psychosis Prevention and Intervention Centre, were aged between 16 – 30 years, and were diagnosed with psychosis within 2 weeks of admission according to the DSM-III-R based on medical record review and either the Royal Park Multidiagnostic Instrument for Psychosis or the Structured Clinical Interview for DSM-III-R. All subjects were screened for comorbid medical and psychiatric conditions by clinical assessment and physical and neurological examination. Exclusion criteria were a history of significant head injury, seizures, neurologic diseases, impaired thyroid function, steroid use, or DSM-III-R criteria of alcohol or substance abuse or dependence. Control subjects with a personal or family history of psychiatric illness were excluded. Clinical information was obtained from patient interview and medical record review. First-episode patients were neuroleptic naïve prior to admission but had received antipsychotic medication prior to scanning. Medication doses for patients with chronic schizophrenia were calculated for the 30 days prior to scanning. FEP cases were followed-up at a 10-year follow-up point (Tables S1/S2).

## Image Acquisition

## PHENOM Model Development Samples

## USA (Pennsylvania)

Structural images were acquired on a 3T TIM TRIO scanner (Siemens, Erlangen, Germany) using T1-weighted 3D magnetization-prepared rapid acquisition with gradient echo sequences (MPRAGE) using the TR = 1810 ms, TE = 3.51 ms, TI = 1100 ms, flip angle = 9 degree, FOV = 240 mm x 180 mm, matrix = 256 × 192, slices = 160, and slice/skip thickness = 1 mm/0 mm.

## Germany (Munich)

T1-weighted MPRAGE images were acquired on a 1.5 T Magnetom Vision scanner (Siemens, Erlangen, Germany) using the TR = 11.6 ms, TE = 4.9 ms, FOV = 230 mm, matrix = 512 x 512, 126 contiguous axial slices of 1.5 mm thickness, and voxel size = 0.45 x 0.45 x 1.5 mm.

## China (Tianjin)

Images were acquired on a 3T MR system (Discovery MR750, General Electric, Milwaukee, WI, USA). Sagittal 3D T1-weighted images were acquired using a brain volume sequence (BRAVO) with the following parameters: TR = 8.2 ms, TE = 3.2 ms, TI = 450 ms, flip angle = 12 degree, FOV = 256 mm x 256 mm, matrix = 256 x 256, slice thickness = 1 mm, no gap, and 188 sagittal slices.

## PHENOM Model Application Samples

## Sao Paulo (PSYCLASS)

For the PSYCLASS sample, imaging data were acquired both at baseline and at follow-up using two identical 1.5-T MRI Scanners (GE Signa, General Electric, USA). The same acquisition protocol was used for all scans: a T1 spoiled gradient recall (SPGR) sequence providing 124 contiguous slices, voxel size 0.86x86x1.5 mm, echo time 5.2 ms, resolution time 21.7 ms, flip angle 20 degrees). Because images were acquired using two MRI scanners, a reliability measure was obtained, as described previously^2, 3^: in brief, six healthy volunteers were scanned twice in each scanner and intraclass correlation coefficients obtained were higher than 0.9 for the neocortical and medial temporal regions.

## Sao Paulo (ESNA)

For the ESNA sample, all participants underwent MRI scanning using a 1.5T Siemens Espree system (Siemens, Erlangen, Germany). Data were acquired using a T1-weighted magnetization-prepared rabid gradient echo sequence (MPRAGE) using the following parameters: TR=2,400ms; TE=3.65ms, NEX=1, field of view=240mm, flip angle=80, matrix=192x192 pixels, slice thickness=1.2mm (no gap between slices), voxel size=1.3x1.3x1.2mm, resulting in 160 slices.

## London

MRI scans were obtained within a 3-month period following the first contact with psychiatric services. A 3 Tesla GE (General Electric, Milwaukee) Signa HDx scanner at the Centre for Neuroimaging Sciences (CNS), Institute of Psychiatry, Psychology and Neuroscience (London, UK) was used to acquire 3-dimensional MPRAGE volumetric scans (matrix size of 256 × 256 × 166 voxels, with in-plane voxel size of 1.02 × 1.02 mm and a slice thickness of 1.2 mm (echo time/repetition time/inversion time = 2.848/6.988/650 ms, excitation flip angle 20°, one data average).

## Santander

Participants were scanned using a 1.5T Signa; General Electric Medical Systems, Milwaukee, Wis) at the University Hospital of Cantabria (Santander). A 3-dimensional T1-weighted spoiled gradient recalled echo image in the steady-state sequence was acquired in the coronal plane. Imaging parameters were as follows: flip angle, 45°; repetition time, 24 ms; echo time, 5ms; voxel dimensions, 1.02x1.02x1.50mm.

## Melbourne

Participants were scanned using a 1.5T Signa; General Electric Medical Systems, Milwaukee, Wis) at the Royal Melbourne Hospital. A 3-dimensional volumetric spoiled gradient recalled echo in the steady-state sequence generated 124 contiguous, 1.5-mm coronal sections. Imaging parameters were as follows: echo time, 3.3 milliseconds; repetition time, 14.3 milliseconds; flip angle, 30°; matrix size, 256 x 256; field of view, 24 x 24-cm matrix; and voxel dimensions, 0.938x0.938x1.5mm. Head movement was minimized by using foam padding and Velcro straps across the forehead and chin. Each scanner was calibrated fortnightly using the same proprietary phantom to ensure stability and accuracy of measurements.

## Preprocessing and MUSE segmentation

A set of extensive quality assurance procedures were applied using both manual verification and automated flags. Raw T1-images were manually examined for motion, image artifacts, or restricted field-of-view. Images were corrected for magnetic field inhomogeneity^10^ and a multi-atlas, multi-warp segmentation method (MUSE)^11^ was used to segment each individual’s images into anatomical regions of interest (ROIs) consisting of gray matter (GM), white matter (WM) and cerebrospinal fluid (CSF). The voxel-wise regional volumetric maps^12^ were generated for GM, WM and CSF tissues by registering skull-stripped T1-images to a template residing in the MNI-space using a deformable registration method for all sites and all subjects^13^. By design, this method utilizes an ensemble of atlases coming from different scanners, field strengths, and acquisition protocols, which renders the method quite robust to such confounds (see ^14^ on comparison with other methods, such as Freesurfer). The processed images were also manually evaluated for pipeline failures, such as for poor brain extraction, poor tissue segmentation, and registration errors. Furthermore, automated procedures flagged images based on outlying values of quantified metrics (i.e., regional volumes) and those flagged images were re-evaluated.

## Inter-site image harmonization and correction of covariates

During model development we previously validated our harmonization approach and schizophrenia subtypes by performing site-wise reproducibility analysis (where the same subtypes were found in each site separately)^1^. Moreover, leave-one-site-out (LOSO) reproducibility analysis was also used, where each site was left out for testing and HYDRA was trained in the rest of sites and this process was repeated in all combinations of sites, and the same subtypes were found. Importantly, in this previous study, proportions of individuals were not significantly different between sites^1^. In the present study, we implemented the approach from our previous study^1^ that we have shown is effective in other clinical and population samples^1^.

Our harmonization approach used regression-based statistical harmonization models and estimated site effects by accounting for site indicators (Figure below) using our original PHENOM development dataset as a reference. This was to: 1) deal with the remaining systematic error on the top of the processing steps involved in the MUSE and voxel-wise regional volumetric maps pipelines; 2) allow explicit estimates of site differences as well as other covariate effects. Analyses indicated no differences between the variances in the scales of the ROI volumes between our original PHENOM data and the current FEP data (Brown Forsythe’s test, p = 0.80), but only in the mean shifts. Hence to be consistent with our earlier studies by Chand et al. ^1, 15^ so that the subtype models could be applied adequately to the first-episode patients to estimate SZ signature expressions, we performed a linear correction that only accounts for additive site effects as in our previous work. By using this linear correction technique with the goal to harmonize to the model development cohort ^1^, we found no difference between the original and current samples (Figure S1) or between healthy controls across sites (Figure S2). Further analyses within single sites showed that the SG1 and SG2 patterns were replicated (Figure S6). These results suggest that scanner protocols were controlled in analyses and thus the observed differences in subgroup proportions are likely to be due to the large clinical differences across samples (Table S4).


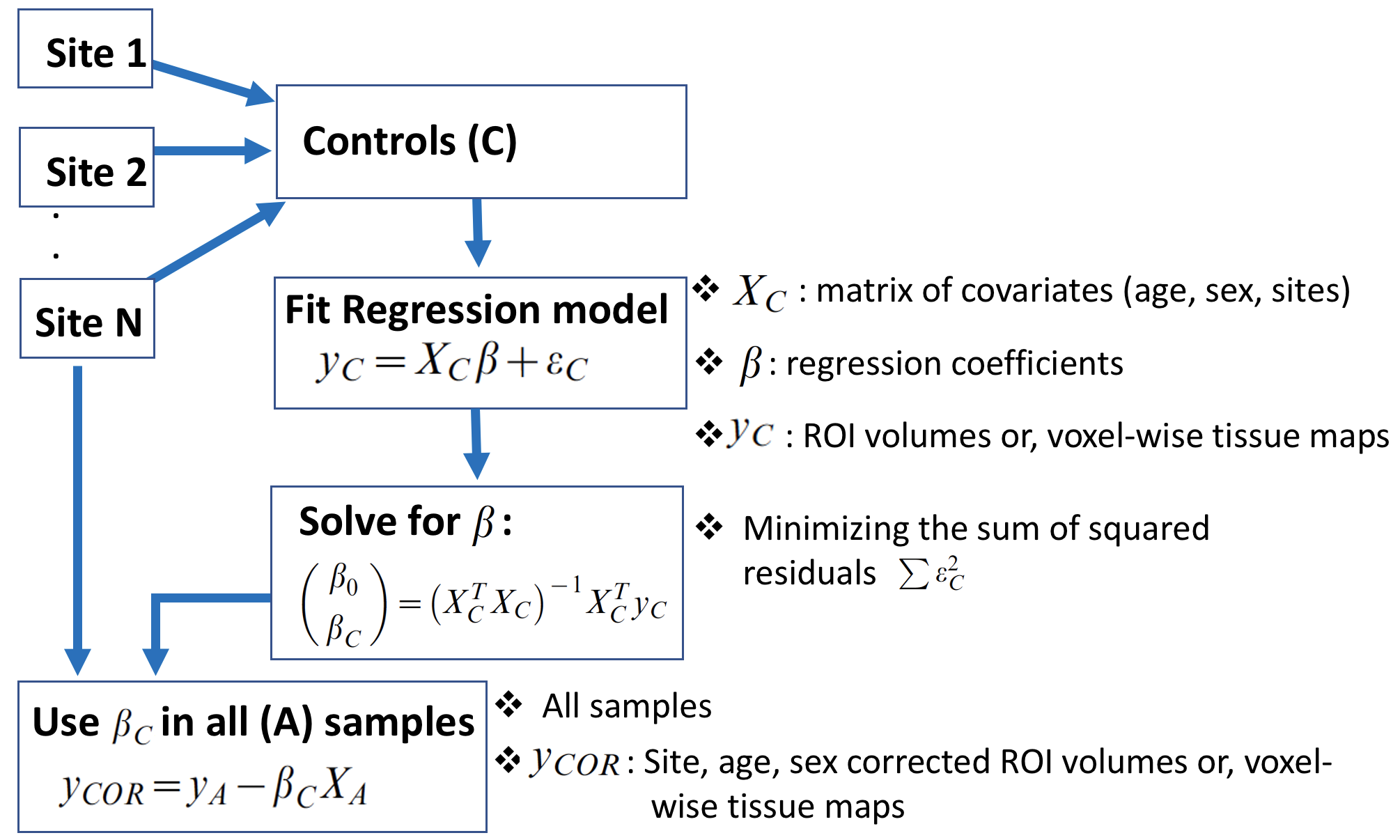


**Schematic of harmonization steps**: Regression coefficients for covariates (age, sex, sites) are computed from controls of all sites and then applied to data from each site to obtain the harmonized ROI volume or voxel-wise tissue (GM or WM) maps.

## Model Development in Established Schizophrenia Sample and Application to FEP Sample: HYDRA

The following details are reproduced from our previous papers^1, 15^. Schizophrenia neuroanatomical dimensional model parameters—weight (W) and bias (b) parameters—were estimated from the original PHENOM Model Development sample^1^ using HYDRA^16^. HYDRA parses disease heterogeneity by clustering cases through their associations with different faces of a polytope relative to the controls created with L2-regularized support vector machines. HYDRA initializes the assignments of cases into clusters by sampling K-unit length hyperplanes considering the space of pairwise differences between cases and controls. The K-hyperplanes are sampled using determinantal point processes^17^ and are used to estimate initial clustering assignments (S^-^). While the estimated solution may vary depending on the initialization, a multi-initialization strategy is implemented. The final clustering results are accomplished based on a consensus of clustering solutions. The HYDRA algorithm can be summarized as:

**Input:** X ∈ R^n x d^, Y ∈ {-1, +1}^n^ (training signals with ‘n’ participants, ‘d’ imaging features), K (number of subtypes)

**Output:** W ∈ R^d x K^, b ∈ R^1 x K^ (classifier); S^-^ (clustering assignment)

**Initialization:** Initialize S^-^

**Loop:** Repeat until convergence (or a fixed number of iterations)

Fix S^-^, solve for W and b

Fix W and b, solve for S^-^

The above analysis was carried out using 50 iterations between estimating hyperplanes and cluster estimation, 20 clustering consensus steps, 0.25 regularization parameter, and 10-fold cross validation. Once the final clusters for two neuroanatomical dimensions were identified, the clusters were fixed, and the optimal W and b were computed. These optimized models were then applied to the preprocessed (MUSE)^11^ and corrected (site, sex, age) MRI first-episode psychosis data from the Sao Paulo PSYCLASS, Sao Paulo ESNA, London, Santander, and Melbourne sites to determine the extent to which each individual expressed the S1 and/or S2 signature.

## Voxel-wise subgroup comparisons: MIDAS

The voxel-wise volumetric profiles between the groups were assessed using MIDAS ^18^ for visualization purposes of signatures. MIDAS parses any set of image maps using a sufficiently large set of overlapping neighborhoods (P), and performs regional discriminative analysis based on least squares support vector machines, which aims to relate the imaging features X ∈ R^n x m^ (‘n’ participants and ‘m’ dimensional imaging features) with group variable Y ∈ R^n^ via a weight vector (w). The regional volumetric pattern that optimally discriminates between groups is equivalent to filtering locally by an optimal kernel whose coefficients are the weights of the discriminant.

# Supplementary Results

## Investigation of medication effects

***Previous work****.* As discussed in the introduction and methods, the current paper applied brain subgroup models that were originally generated in a chronic sample (N=307) to first-episode cases. In this previous work, the question of whether antipsychotic effects confounded the results was highly relevant because of the longer duration of illness. In that previous study, we tested this by analysing the relationship between chlorpromazine equivalent antipsychotic dose and the antipsychotic type. Both tests were non-significant (dose: T(210)=-0.44, p=0.521; type: χ^2^(3)=6.67, p=0.083). As we noted in that publication, the importance of structural heterogeneity that is not attributable to medications or chronicity is supported by evidence that cortical reductions and basal ganglia enlargement may occur in medication-naïve populations with clinical and genetic risk ^19-22^.

As reported in the introduction (Main Text), we also recently published a paper investigating the presence of the subtypes found in chronic cases in large population samples (Philadelphia Neurodevelopmental Cohort, N=359, ages 16-23yrs; UK Biobank, n=836, ages 44-50yrs). In that study, both samples were unmedicated and we replicated the two neuroanatomical signatures. We further found that Signature 1 (i.e., reduced brain volume) was more common in youths with psychosis spectrum symptoms and associated with schizophrenia polygenic risk, whereas Signature 2 was not. When combined, our previous results using the same brain subtypes that were used in this study to separate first-episode patients suggest that antipsychotic usage does not mediate group membership and is associated with the intrinsic structure of the brain.

***Current work (existing analyses)***. Our previous evidence strongly suggested that antipsychotic usage was not mediating the presence of subgroups in the current study. To further support this hypothesis, in the current study we followed the methodology of our previous work in population samples and applied the subtype models to our healthy control sample. These results replicated the previous finding showing the presence of signature 1 and signature 2 in healthy controls who are not treated with any psychoactive medication (Figure 1, main text). As reported in the supplementary materials (Figure S3), we also conducted voxel-wise analyses only in the healthy control sample to find similar patterns of brain volume reductions in SG1 and increased striatum in SG2. As we reported, we also replicated our previous results^1^ in finding that the prevalence of signature 1 was higher in first-episode psychosis patients compared to controls whereas there was a similar proportion of signature 2 cases across study groups.

***Current work (new analyses)***. Despite the evidence that antipsychotic effects were not solely mediating subgroup membership across multiple analyses in patients and healthy controls, we sought exclude the influence of antipsychotics on the primary findings in the first-episode sample. We firstly acquired additional data to assess differences in the CPZ dose, antipsychotic type, and the duration of antipsychotic treatment (estimated by calculating the difference between the total duration of untreated psychosis and the duration of psychosis). We then described differences in the subgroup samples and performed three main corrections related to each of our primary analyses: 1) we regressed antipsychotic dose and type (typical versus atypical) from the region-of-interest volumes (along with site, age, and sex) and determined the subgroup labels; 2) we regressed antipsychotic effects from the voxel-wise analyses and visualised differences; 3) we controlled for antipsychotics in clinical baseline and follow-up analyses.

Results of the association between antipsychotic type and subgroup are displayed in Table R1. Antipsychotic class indicated significant differences between the subgroups reflecting an increased usage in the SG1 subgroup compared to the ‘None’ and SG2 (p<0.05) in addition to an increased typical usage in the SG1+SG2 subgroup compared to the SG2 subgroup (*p*<0.05). This difference was in the context of a small total number of cases who were taking typical antipsychotics (57, 6% of the sample). Results also demonstrated reduced CPZ-equivalent dose of the ‘None’ subgroup compared to all other subgroups (*p*<0.05) and increased dose of the SG1+SG2 subgroup compared to SG1 (*p*<0.05); no differences were found between the SG1 and SG2 subgroups.

As missing antipsychotic data was identified, we used two approaches in the following analyses to thoroughly exclude the effects: 1) imputation of antipsychotic data using the baseline clinical features included in the main analyses (Main Text; knn 7-nearest neighbours); 2) exclusion of individuals with missing antipsychotic data.

Regression of antipsychotic effects (type and CPZ-dose) prior to application of the models revealed similar results (Figure S5). Using imputed data, we found that the correlation between the membership scores (i.e., those depicted in Figure R7 below) was 0.98 (*p*<0.001) for SG1 and 0.99 (*p*<0.001) for SG2. We then defined the subgroup labels using the method described in the Main Text and used the Adjusted Rand Index to determine the similarity between the subgroup assignment (0-1 with 1 representing perfect assignment; Rand=0.91). We finally directly compared the proportions of cases in each subgroup (Figure S5) and verified that the proportional differences to healthy controls were maintained (e.g., in the SG1 subgroup; Chi^2^=49.51, *p*<0.001). Apart from a minimal decrease of SG2 proportions in the FEP sample (20.6% uncontrolled / 17% controlled), most likely due to borderline cases, these results suggest that control of antipsychotics results in minimal changes in membership scores and subgroup assignments. Replication of results using data with cases excluded if they did not contain antipsychotic data were highly similar (SG1 score r=0.98, p<0.001; SG2 score r=0.99, p<0.001; Rand=0.90; Chi^2^=50.16, *p*<0.001; Figure R7).

We then controlled for antipsychotic type and dose in brain analyses by linearly regressing the effects prior to MIDAS analysis (Figure S5, below). Results showed that the pattern of widespread reductions in cortical/subcortical volume in the comparison of the SG1 versus “None” subgroups remained (Figure S5). The pattern of increased striatal volume was also retained in the comparison of “None” versus SG2 subgroups. These results agree with our previous analyses in chronic and population cohorts. They support our conclusions that the brain subtypes are present prior to illness onset and are not influenced by antipsychotic use.

We then repeated the clinical baseline analyses of subgroup separation and the follow-up analyses of remission prediction (Figure S11 & S12). At baseline, both approaches revealed similar patterns associated with SG2 membership with education, unemployment, and CPZ-Z dose. The association with positive symptoms was reduced, which is likely to be the effect of unavoidable interactions between antipsychotic dose and positive symptom severity. Prediction of remission across follow-up timepoints also revealed overlapping patterns with the inclusion of SG2 membership at the 1- and 5-year timepoints. At uncorrected levels, SG2 membership was also found when remission was analysed across all timepoints. The results were also replicated when excluding individuals without medication data (Figure S12).

Overall, the results indicate that: a) the brain subtypes are found in the general population across multiple samples; b) controlling for antipsychotic dose and type in subgrouping, voxel-wise, or clinical analyses does not substantially modify the results. When combined, these results support the conclusion that the brain subtypes are present before illness onset and are related to distinct clinical phenotypes and outcomes.

## Investigation of differences in the follow-up population only

We found a range of clinical differences associated with the follow-up sample. We thus compared the subgroups on the previously described battery of measures (Table S9). In similarity to the original analysis, we found increased education in the SG2 subgroup. We also repeated the clinical multivariable analyses predicting remission only in individuals with follow-up data and found a similar pattern, with the only difference that there was less of an association with positive symptoms (Figure S14)—potentially due to decreased symptom load overall in the follow-up sample.

## Elaboration on the neurobiological significance of the two subtypes

In our previous work defining the subtypes^1^ we hypothesised about the neurobiological significance of the two subtypes. We have reproduced these hypotheses here, added to the second hypothesis by considering the influence of sex on SG2 remission, and included a comparison with a recently published subgrouping paper^23^.

**SG1**. The widespread volume decreases in Subtype 1 are consistent with mechanisms associated with early neurodevelopmental disruption, inflammation, and cortical dysfunction, where exaggerated activity of the complement-microglia system can produce synaptic over-pruning and impair interneuron migration^24, 25^. Interneuron dysfunction, and aberrant cortical development more broadly, is also linked to hyperglutamatergia^26^ and disrupted excitatory/inhibitory balance in cortex^26, 27^. While these mechanisms could result in secondary dopaminergic disruption^28^, the existence of primary non-dopaminergic abnormalities could render subtype 1 less responsive to current dopamine-blocking antipsychotics. As such, the lack of a relationship between SG1 and remission could be related to these mechanisms along with the increased baseline prevalence of first-generation antipsychotic usage.

**SG2**. The selective striatal increase in SG2 is notable given the high concentration of dopamine and D2/3 receptors in this region^29, 30^ and increasing evidence in medication-naïve, clinical-risk, and genetic-risk psychosis populations indicates striatal hyperdopaminergia^28^ and larger basal ganglia^31-34^. Schizophrenia polygenic-risk and single-risk alleles also associate with larger putamen in non-clinical samples^21^. Further, a recent study found increased putamen volume in a transdiagnostic medication-naïve sample and unaffected-family members^20^. In combination, our findings may suggest a primary hyperdopaminergia schizophrenia subtype that has not been previously detected. Interestingly, we also found that SG2 membership and female sex were related to remission. This finding agrees with previous top-down research suggesting increased striatal volume is related to remission in subgroups of female individuals with psychosis^35, 36^. Research also demonstrates that even in healthy control populations females exhibit differences in striatal dopaminergic synaptic concentrations and receptor functioning^37^. Additionally, lower antipsychotic doses are required in females to achieve the same D2 receptor occupancy compared to males^38^. When combined, the increased striatal volume in SG2 females could be reflective of baseline sex differences related to hyperdopaminergia mediated by baseline differences in synaptic concentrations and functioning. Further research is required to investigate this hypothesis.

**Comparison with Zhao et al. (2022)**. In a recently published cross-sectional paper, Zhao et al. (2022)^23^ used unsupervised learning (k-means) on 14 subcortical volumes from a discovery cohort of institutionalised patients with chronic schizophrenia. Two subgroups were found: 1) SG1 characterised by widespread volume reductions of both subcortical and cortical volumes; 2) SG2 characterised by increased pallidum volume with localised volume reductions in frontal, temporal and parietal lobes. Supervised learning was then used to identify the subgroups in a drug naïve first-episode schizophrenia (FES) sample and a sample from the Bipolar-Schizophrenia Network on Intermediate Phenotypes (B-SNIP). SG1 validation results demonstrated a similar direction of effect in both samples, but significant differences were not found for cortical volume reductions in SG1. SG2 validation results exhibited increased pallidum in the B-SNIP sample, but not in FES, which instead demonstrated increased striatal volumes compared to healthy controls. Increased putamen volume and cortical volume reductions in SG2 were also detected in the B-SNIP sample. The authors’ interpreted the SG1 findings in relationship to illness progression in some individuals, antipsychotic mediation effects, or a distinct subpopulation of patients with the features from the illness onset. SG2 findings were interpreted in relationship to literature demonstrating higher pallidum volume in cases treated with antipsychotics, but not in those who are untreated.

As the authors’ write, their results are broadly consistent with our clusters from Chand et al. (2020) because both studies found two subgroups: one with cortical-subcortical decreases and the other with increases of basal ganglia structures. However, some key differences are important to note. In relationship to SG1, rather than being restricted to chronic cases, we found volumetric decreases within chronic^1^, first-episode (current paper), and population-based samples^15^. For SG2, rather than finding increased pallidum with localised cortical reductions, we also found increased striatal volume across the same samples and did not find cortical differences. Such differences between studies could be related to the clustering targets and levels of severity within the discovery samples used to define the subgroups: in Zhao et al. (2022) subcortical clustering of patients with a mean illness duration of 20-years, whereas Chand et al. (2020) employed whole-brain clustering in an international community sample with a mean illness duration of 7-years. Sample differences could have resulted in the identification of broad subgroups representing schizophrenia vulnerability axes in Chand et al. (2020), which are present across samples, whereas, more specific subgroups related to longer-term illness could have been detected in Zhao et al. (2022).

Despite the differences between the studies, the subgroup formulations are likely to overlap and share similar neurobiological mechanisms. For example, a subset of individuals either from the SG2 or SG1+SG2 subgroups in the submitted study who are vulnerable due to hyperdopaminergic states may further develop towards an illness stage represented by the SG2 subgroup in Zhao et al. (2022) with specific pallidum increases and frontal, temporal, parietal decreases (e.g., potentially mediated by pallidal-cortical connections^39^). Similarly, cases in the SG1 subgroup in the submitted study with suspected non-dopaminergic mechanisms (e.g., hyperglutamatergic or excitatory-inhibitory imbalance) may further develop towards a more specific chronic SG1 signature as found in Zhao et al. (2022). Investigating such hypotheses requires two additional analyses: 1) direct comparison of the subgroups from the two studies in the same samples; 2) longitudinal investigation of brain changes over the illness course in both subgroups. Based on the results from Zhao et al. (2022), a further prediction may be that antipsychotic treatment may be associated with further increases of basal ganglia structures and particularly the pallidum in some SG2 or SG1+SG2 individuals—however, it was notable that in Zhang et al. (2022), Chand et al. (2020), and the current study there were no baseline relationships to antipsychotic dose and therefore longitudinal clinical trial comparisons would be necessary.

## Investigation of subgroup separation using unsupervised methods

Our motivation for using the semi-supervised HYDRA approach was to sensitively detect previously unidentified subgroups relative to a healthy control population. In the context of our use of the multi-atlas, multi-warp segmentation (MUSE) technique to derive the features, we reasoned that the use of HYDRA would provide further control over such nuisance variables present in both populations, including sex, age, and scanner site. However, we note that this technique is different to traditional unsupervised clustering techniques used in previous research and it would be interesting to compare the results of both algorithms.

Our original analyses were conducted in a community sample of mostly chronic patients ^1^, thus we used this sample again to generate clustering memberships before learning and applying the solution to the first-episode sample. In the context of a completely unsupervised analysis, the inclusion of healthy controls is an open question and thus we chose to conduct two analyses: 1) inclusion of schizophrenia patients only (n=307); 2) combining both healthy controls and first episode psychosis (FEP) patients (n=671). We employed a k-means++ approach, as used in Zhao et al. (2022), within the MATLAB 2021b environment with squared Euclidean distance and 100 replicates. The 145 age, sex, and site corrected brain MUSE volumes from the original discovery sample paper^1^ were used in the analysis. The Calinski Harabasz, Davies Bouldin, and Silhouette measures determined the optimal number of clusters (majority vote was used in cases of non-agreement). Supervised machine learning employing a Support Vector Machine (SVM; libsvm 3.12) within a 10x10-fold nested cross-validation procedure determined a separating hyperplane dividing the subgroups and the models were subsequently applied to the first-episode sample to determine cluster membership.

1. Results using discovery sample of patients only

When using a discovery sample consisting of patients^1^, results demonstrated a 2-cluster solution wherein 86% of patients in SG1 of the k-means++ solution were also included in SG1 of the original semi-supervised solution of Chand et al. (2020) and 63% of the patients in SG2 were also derived from the original SG2 division (Table S11). Comparison of the brain maps revealed a similar distribution of lower grey matter in SG1 when compared to the healthy control group (Figure 15). The pattern of brain differences in SG2 when compared to controls was also similar, but exhibited reduced effect size and spatial extent (especially of striatal regions; Figure 15).

Application of the k-means++ subgroups to the first-episode sample revealed 40% of the original SG1 cases in the kmeans++ SG1 subgroup and 34% of the original SG2 cases in the kmeans++ SG2 subgroup (Table S12). Differences between the subgroups were found across all 4 sites, indicating a substantial site effect—i.e., greater than the original semi-supervised solution of Chand et al. (2020) with site effects in 2 sites. Diagnostic differences were detected, with more schizophreniform and schizoaffective cases in the k-means++ SG1 subgroup, which also demonstrated increased symptoms (Table S12). Notably, unlike in the original semi-supervised learning analysis, differences in education were not detected (Table S12). Longitudinal analyses also did not reveal significant differences in remission between the two subgroups (Table R3). For example, the semi-supervised analysis demonstrated increased remission across all years (57% in SG1 versus 78% in SG2; p=0.02) but this was not the case for the k-means++ analysis (66.3% in SG1 versus 66.0% in SG2).

1. Results using a discovery sample with patients and controls

We then repeated the discovery analyses in the original sample ^1^, but included both the healthy controls and the patients in the analysis. The optimal number of clusters was again k=2 based on the Calinski Harabasz and Sillouette measures (Davis Boudin indicated k=4). Application of the models to the first episode sample revealed similar results and no relationship with remission (Tables S14 and S15).

1. Discussion

When combined, the results demonstrate the robustness of the subgroup solution at a broad level when using a completely unsupervised technique (k-means++). This was especially the case for the SG1 subgroup, which was highly replicable across techniques. However, major findings related to SG2 membership were not replicated—i.e., the inclusion of striatal areas in the chronic sample and association with the educational attainment and remission in the first-episode sample. We hypothesise that detection of such differences following semi-supervised analyses may occur because the healthy reference group helps to control for nuisance variance as we outline in the Main Text—e.g., as indicated by increased site differences between the subgroups. Additionally, the semi-supervised technique also allowed the division of new cases into ‘None’ and ‘Mixed’ subgroup classes to further reduce heterogeneity, but could not be detected in the k-means++ analysis (e.g., because we did not find separable healthy control subgroups in analysis 2). When combined, the results demonstrate a need for further research directly comparing the subgroup solutions across studies (e.g., with Zhao et al. 2022), samples, and clustering techniques.

**Figures**


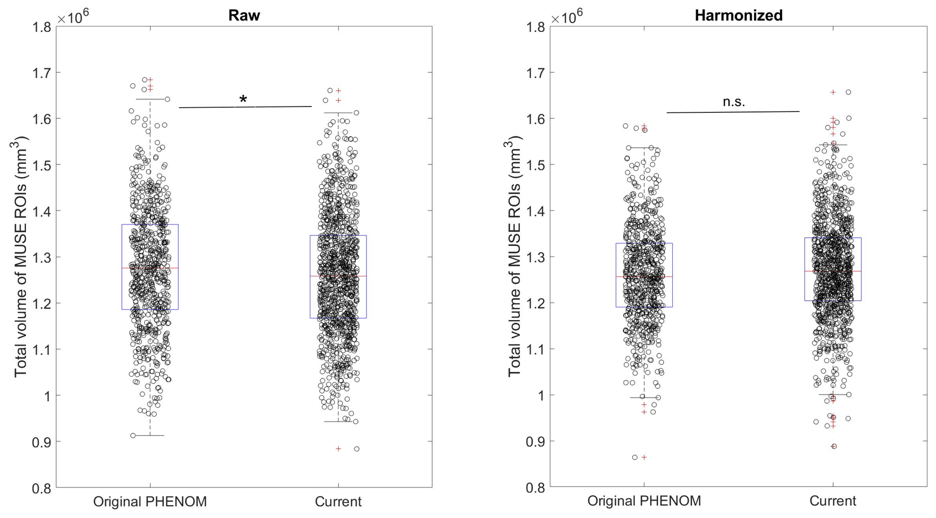


Figure S1. Illustrates raw data and harmonized data where our harmonization steps minimize site differences. The models were developed in the “Original PHENOM” sample and applied to the “Current” first-episode psychosis sample following linear site correction. Raw data had significant site difference (*: p < 0.05), while there is no site difference in the harmonized data.


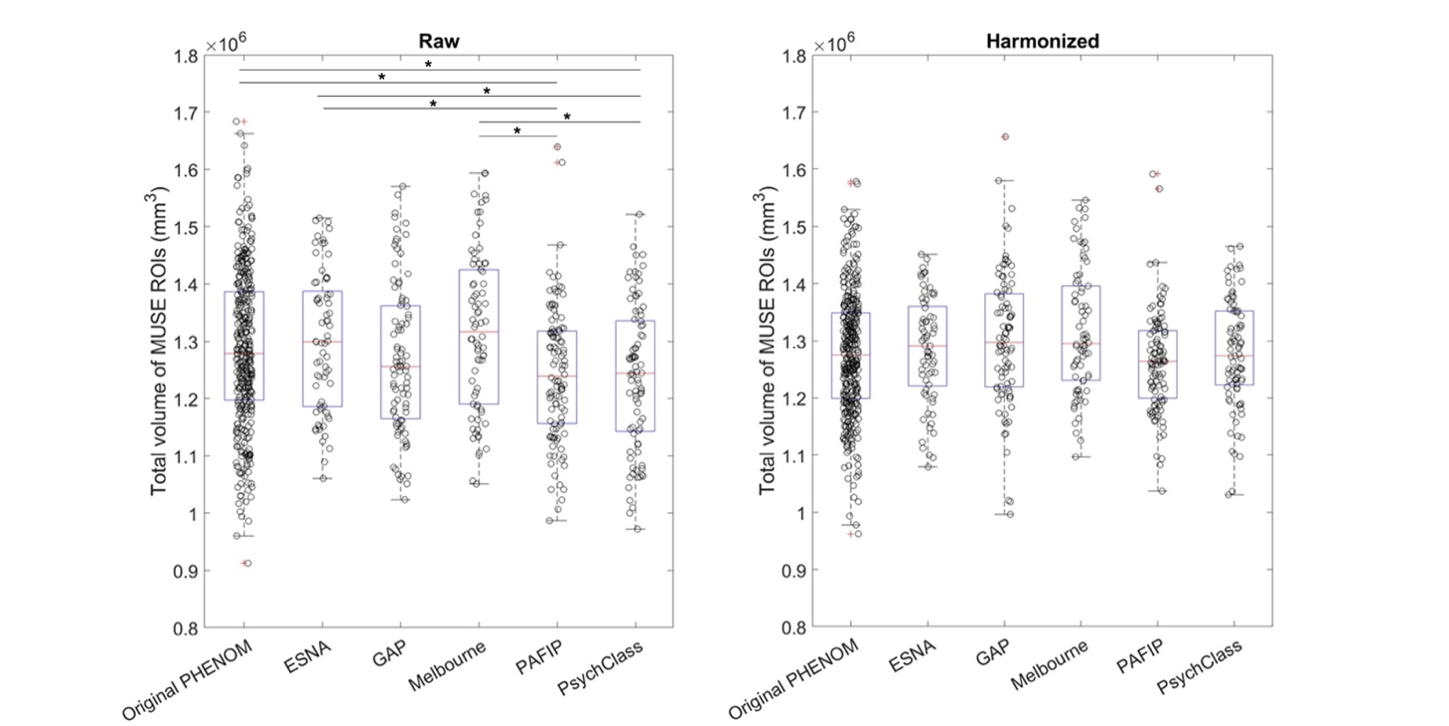


Figure S2**.** Raw data and harmonized data across all sites only in the healthy control population. Multiple site differences (p<0.05) were observed in the raw data, which were not observed following harmonization to the original PHENOM cohort prior to model application.

**
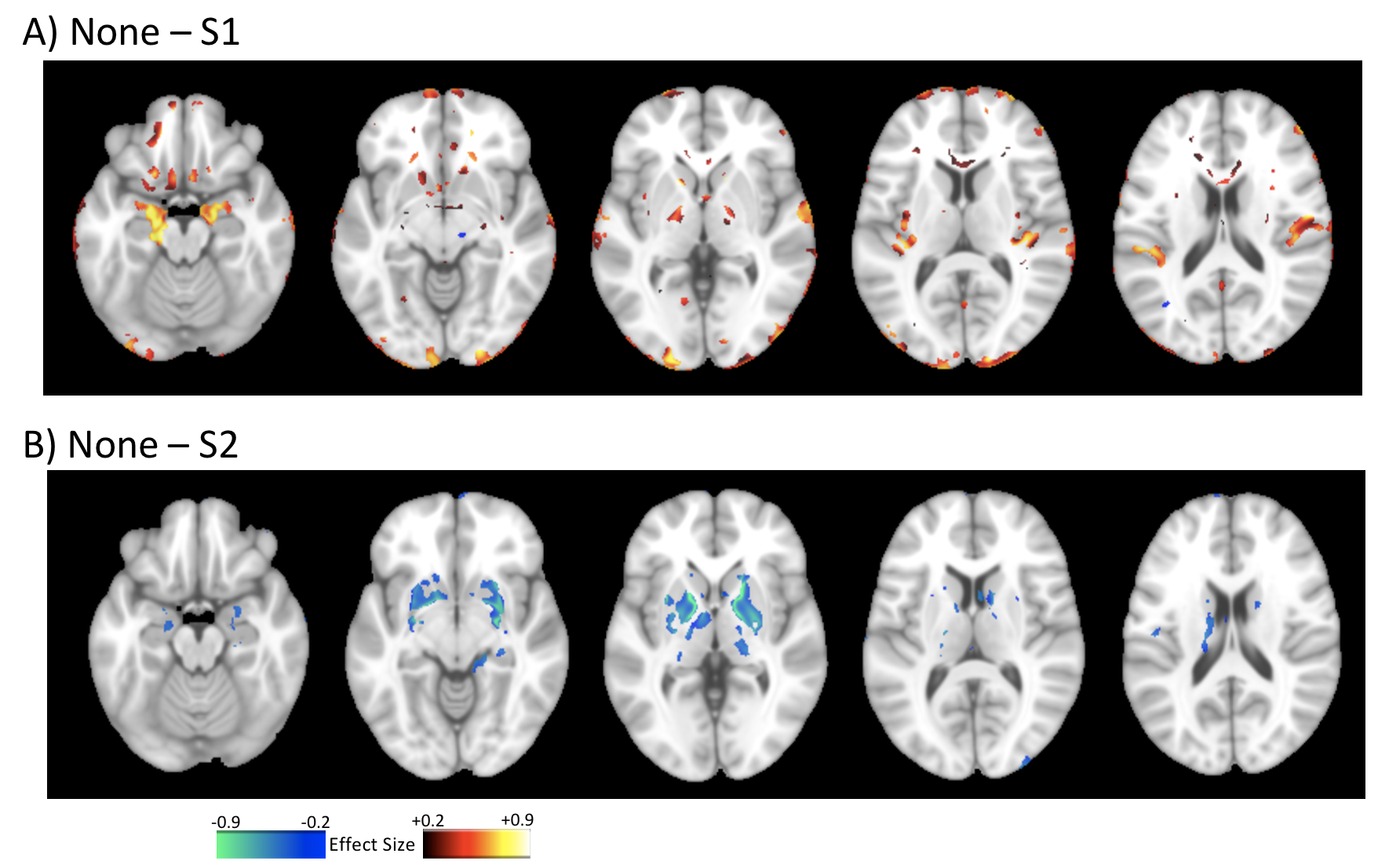
**

Figure S3**.** Voxel-wise differences in healthy control subjects between the “None” brain subgroup and both SG1 and SG2 subgroups. A) differences were distributed throughout the brain and restricted in extent compared to the sample also including the first episode psychosis patients; B) The SG2 subgroup demonstrated increased striatal volume as found in the SG2 subgroup containing first episode patients.

**
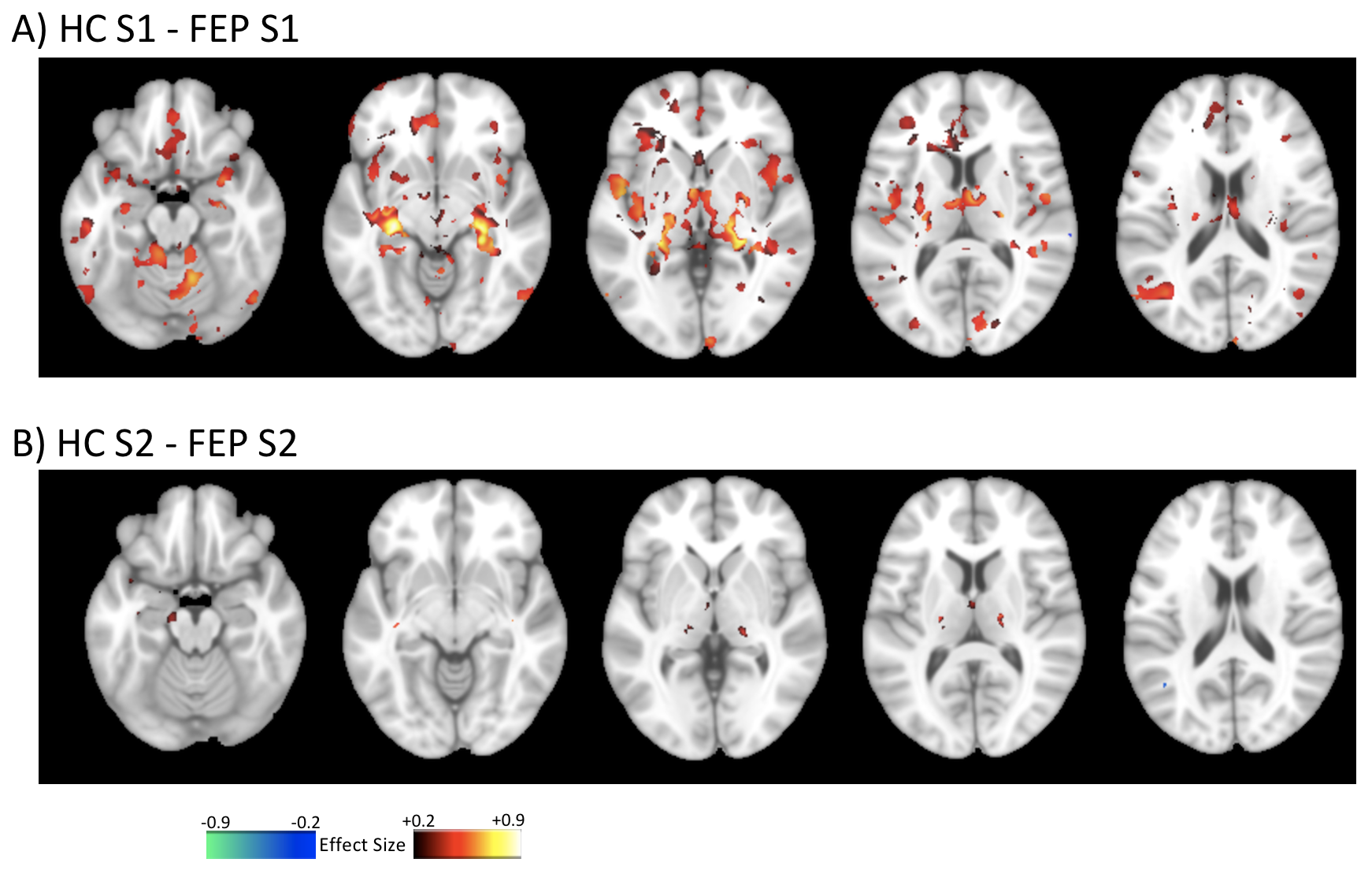
**

Figure S4. Comparison between the healthy control and patient cohorts within each of the subgroups. A) Comparison within the SG1 subgroup demonstrated additional reductions in the first-episode sample. B) Minimal differences (e.g., small thalamic clusters) were found within the SG2 subgroup between healthy controls and first episode psychosis patients.


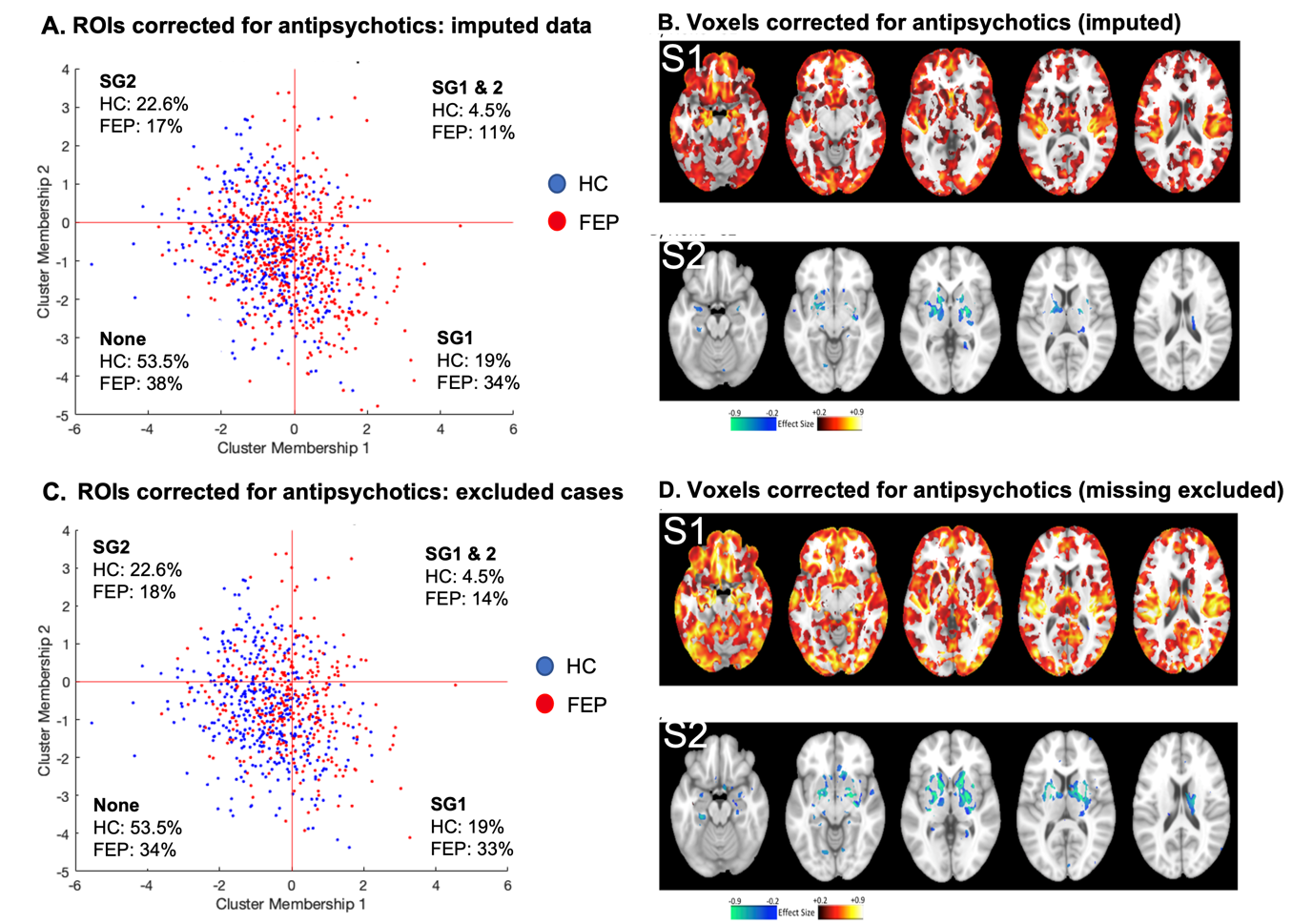


Figure S5. Subgroup and brain differences when controlling for antipsychotic dose (z-scored chlorpromazine equivalent) and antipsychotic type (typical versus atypical). A) Correction of the regions-of-interest (ROIs) using imputed antipsychotic data prior to the application of the subgroup models resulted in similar results to the main analyses (Main Text, Figure 1). B) Voxel-wise data was corrected using imputed antipsychotic data and Cohen’s d effect size maps comparing subgroups with the ‘None’ category were generated by masking MIDAS results after FDR-correction over voxels at p<0.05. Patterns are similar to those without the control of antipsychotics in the FEP sample, in addition to previous analyses in chronic and population-based samples. C/D) Results were maintained when excluding cases without antipsychotic data.


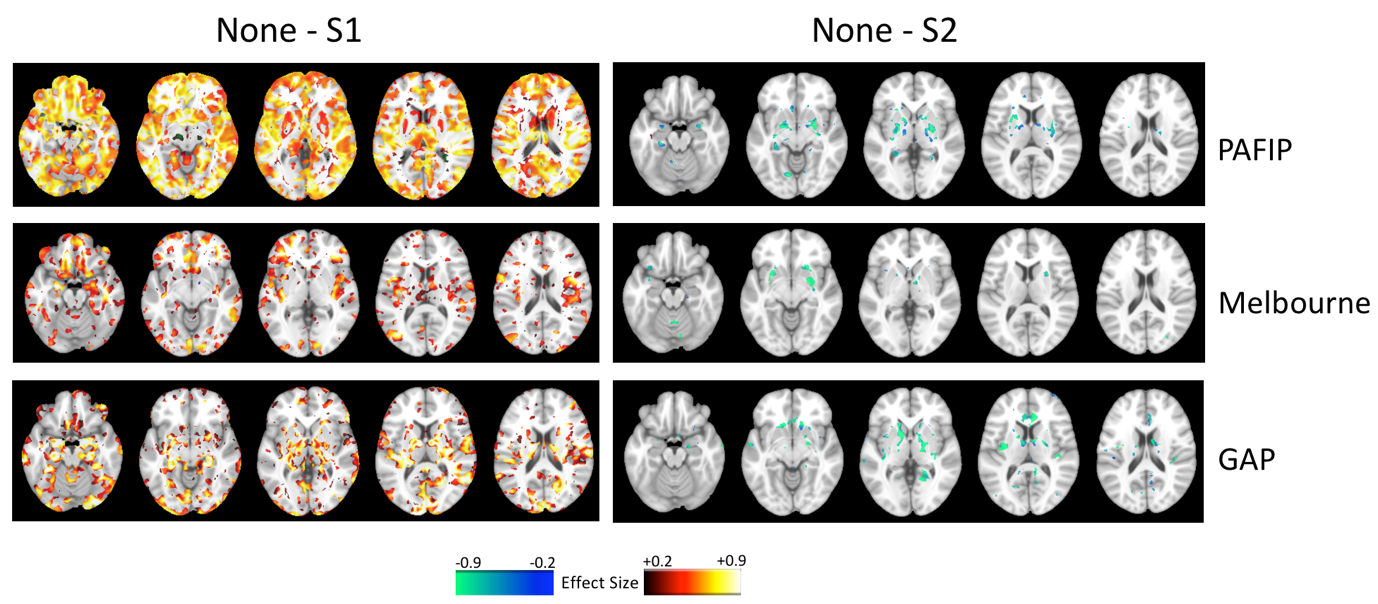


Figure S6. Brain patterns found when investigating effects within sites. Widespread decreases were found in SG1 across sites and increased striatum was found in SG2.


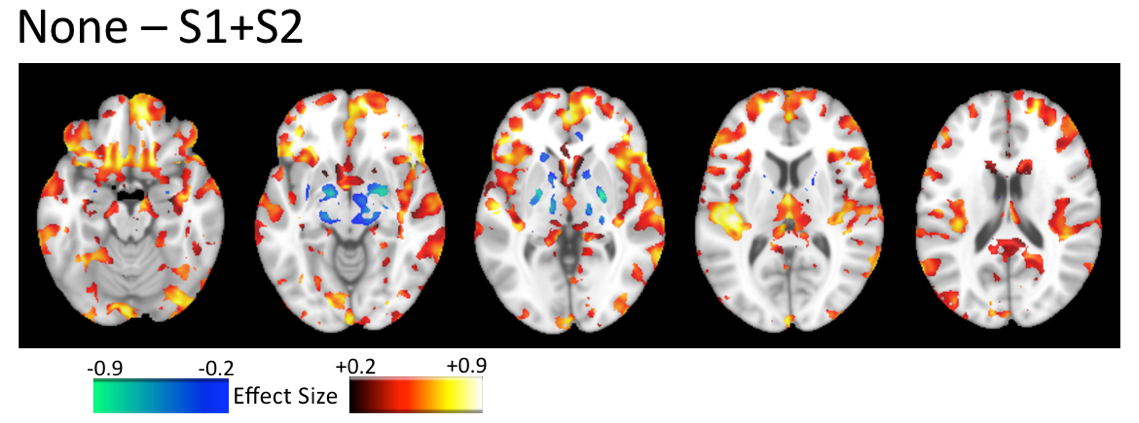


Figure S7. GM comparison of SG1+SG2 compared to ‘None’ in FEP patients showing widespread cortical deficits and very mild increase in striatal volume. Note Cohen’s d effect size maps were generated by masking MIDAS results after FDR-correction over voxels at p<0.05.

**
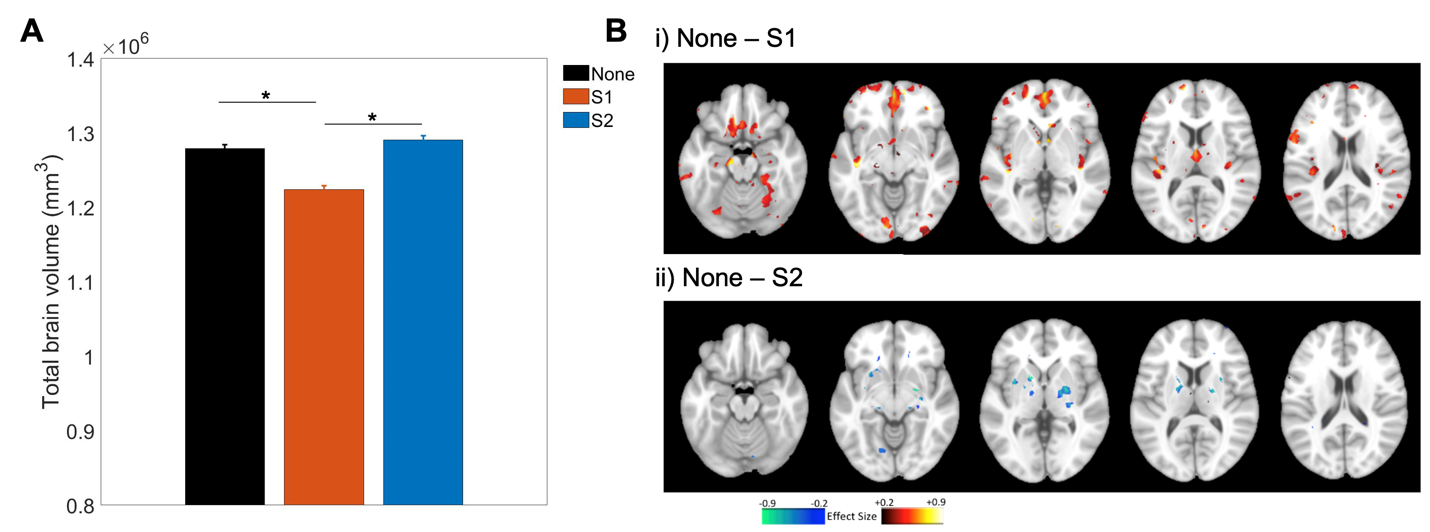
**

Figure S8. Comparison of the brain subtypes after correction for intra-cranial volume (ICV). A) total brain volume remained decreased in the SG1 subgroup following ICV control (*, <0.05). B) distributed decreased brain volume compared to the ‘None’ subgroup was also found in SG1 and increased striatal volume in SG2 compared to ‘None’. Cohen’s d effect size maps were generated by masking MIDAS results after FDR-correction over voxels at p<0.05.


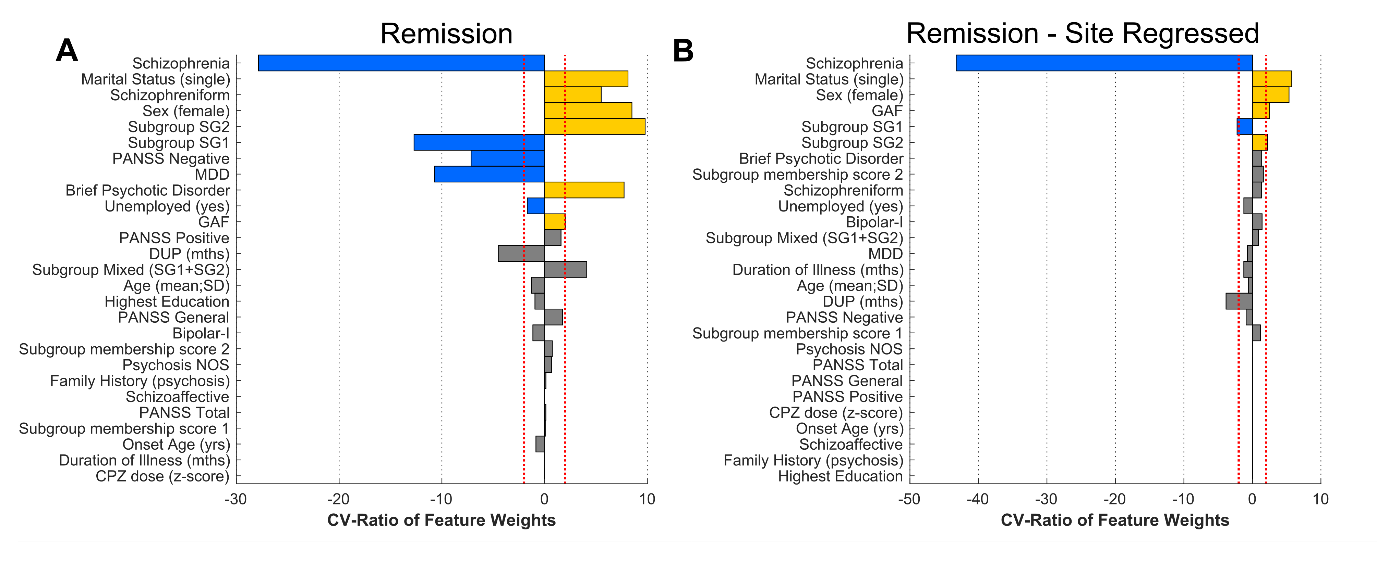


Figure S9. Prediction of remission at any timepoint with no site control (A) and with regression of site (B). **A)** Prediction of remission included increased single marital status, schizophreniform or brief psychotic disorder diagnoses, female sex, and global assessment of functioning (GAF) in addition to reduced schizophrenia diagnoses, negative symptoms, and major depressive disorder. Increased SG2 was associated with remission, while decreased SG1 membership (relative to the other variables in a multivariate analysis), was associated with remission. **B)** Associations with decreased SG1 and increased SG2 membership remained when controlling for site.


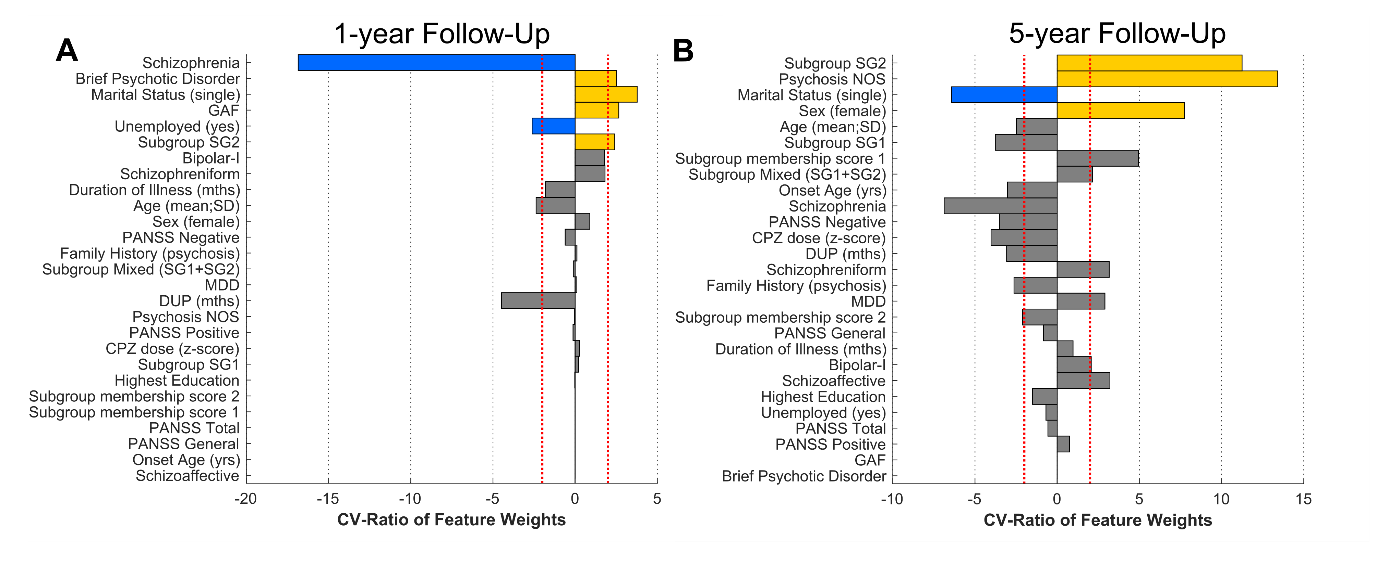


Figure S10. Regression of site within the training samples of the cross-validation routine prior to prediction of remission resulted in signatures that retained the SG2 subgroup label at 1- and 5-years.


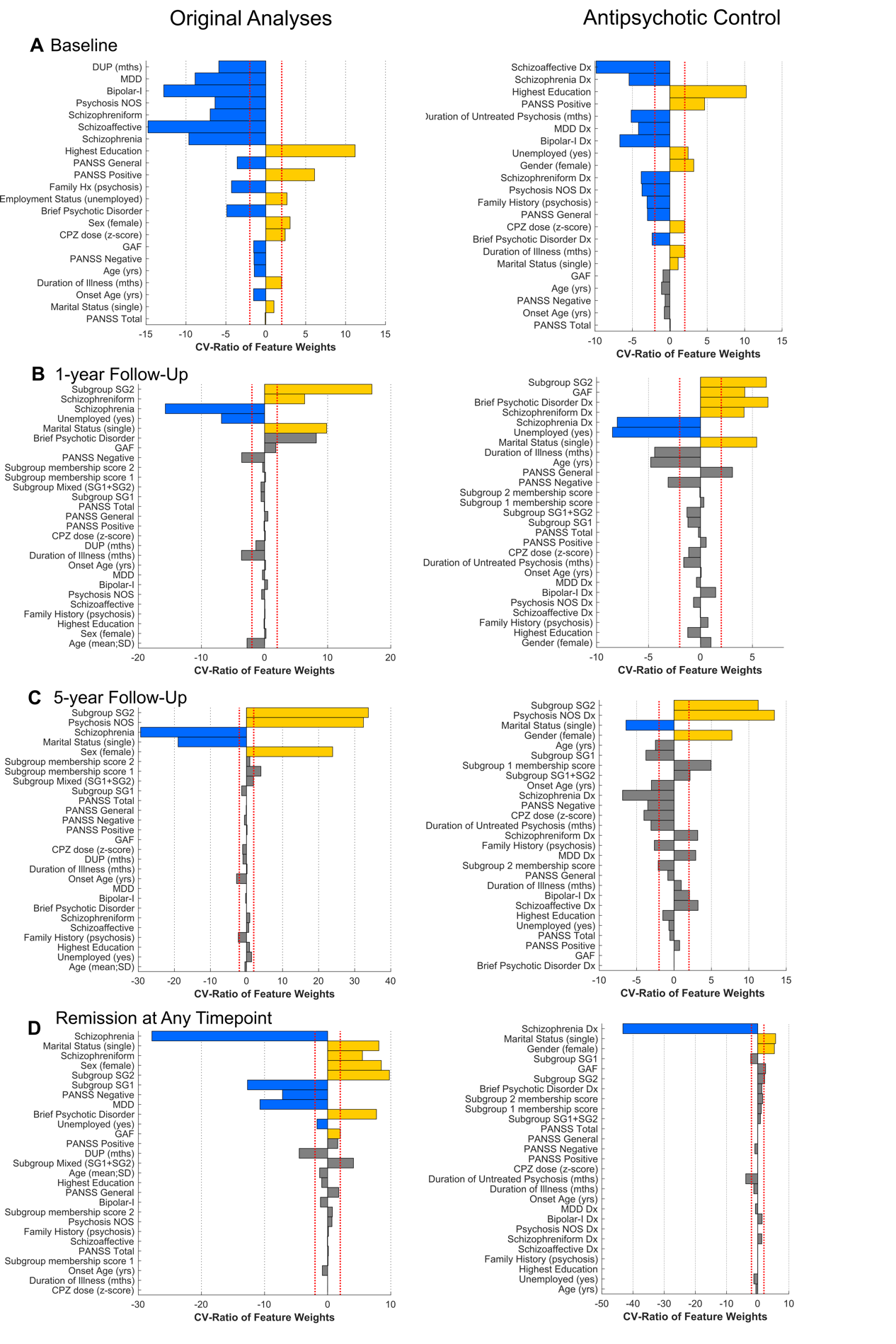
Figure S11. Analysis of baseline and follow-up predictive patterns while controlling for antipsychotic dose (z-scored chlorpromazine equivalent) and type (typical versus atypical). Original results without controlling for antipsychotics are in the left column. Results controlling for antipsychotics are depicted in the right column. Missing data for medication variables was imputed using a nearest neighbour approach using the predictors.


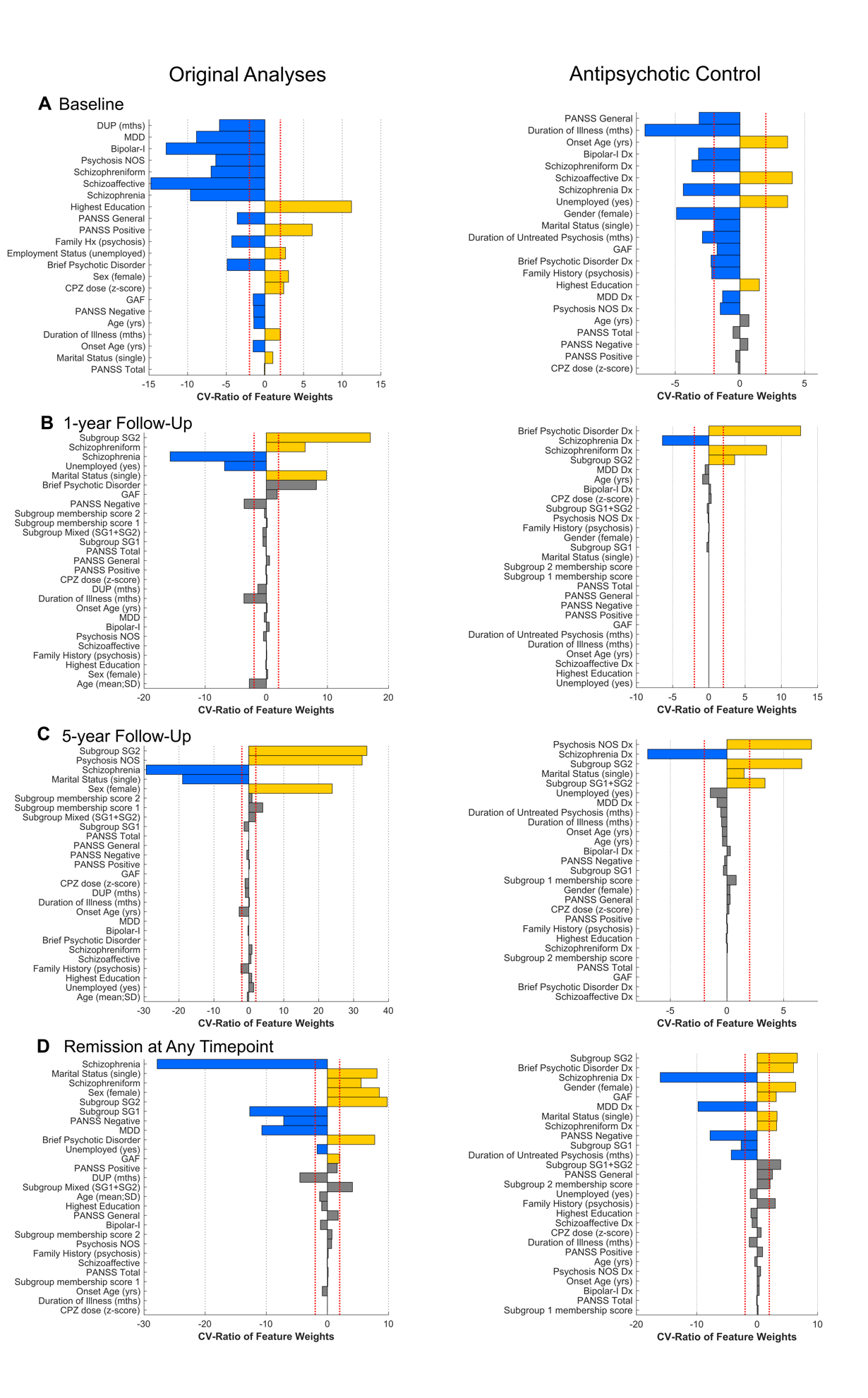


Figure S12. Analysis of baseline and follow-up predictive patterns while controlling for antipsychotic dose (z-scored chlorpromazine equivalent) and type (typical versus atypical). Original results without controlling for antipsychotics are in the left column. Results controlling for antipsychotics are depicted in the right column. Patients excluded if missing data for either antipsychotic variable.


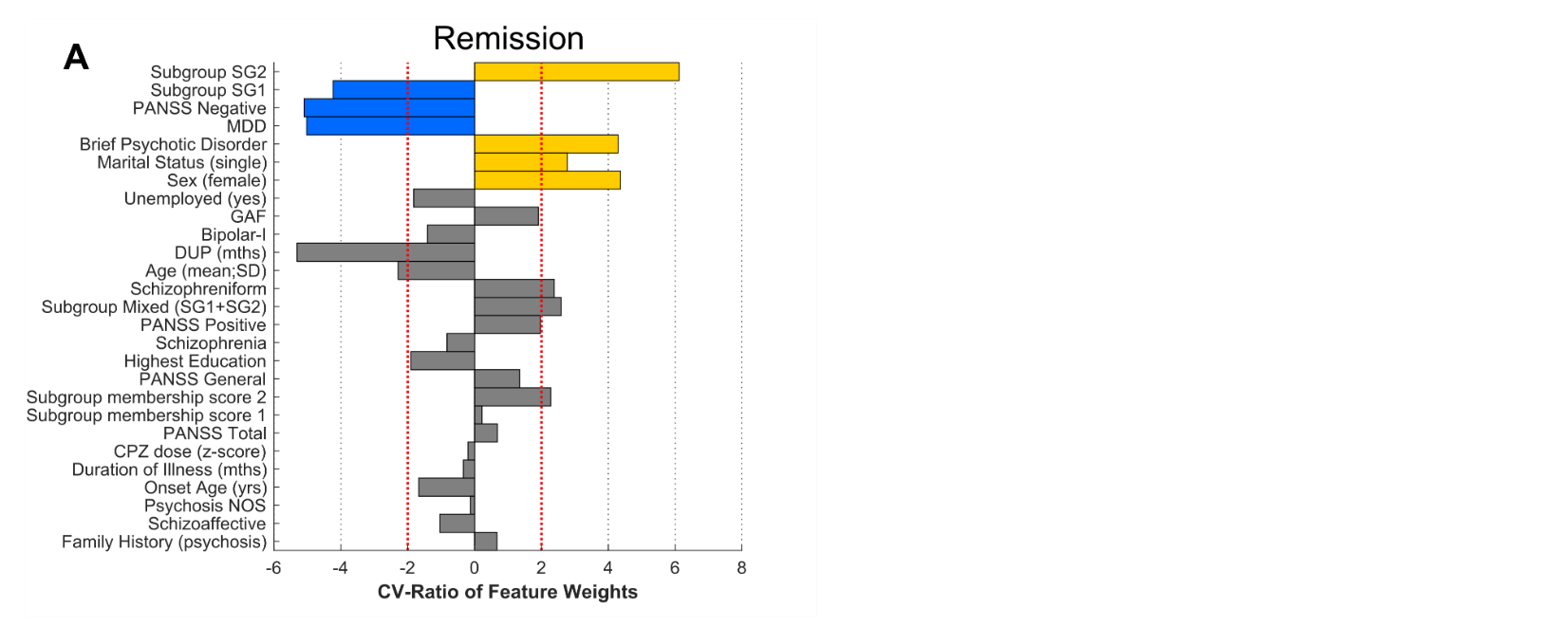


**B**

Figure S13**.** Controlling for a diagnosis of schizophrenia or drug use clarified the relationship with remission in analyses of all timepoints. **A)** At FDR-corrected levels of significance related to the sign-based consistency measure, the remission prediction pattern included increased SG2 membership and decreased SG1 membership. **B)** Controlling for either lifetime marijuana (yes/no) or “other illicit drug use” (yes/no) also clarified the relationship with remission with increased SG2 and decreased SG1 membership.

Figure S14. Baseline comparison of SG1 versus SG2 only in follow-up individuals. A similar pattern of increased diagnoses was found for SG1 (blue) and increased education in SG2 (yellow).


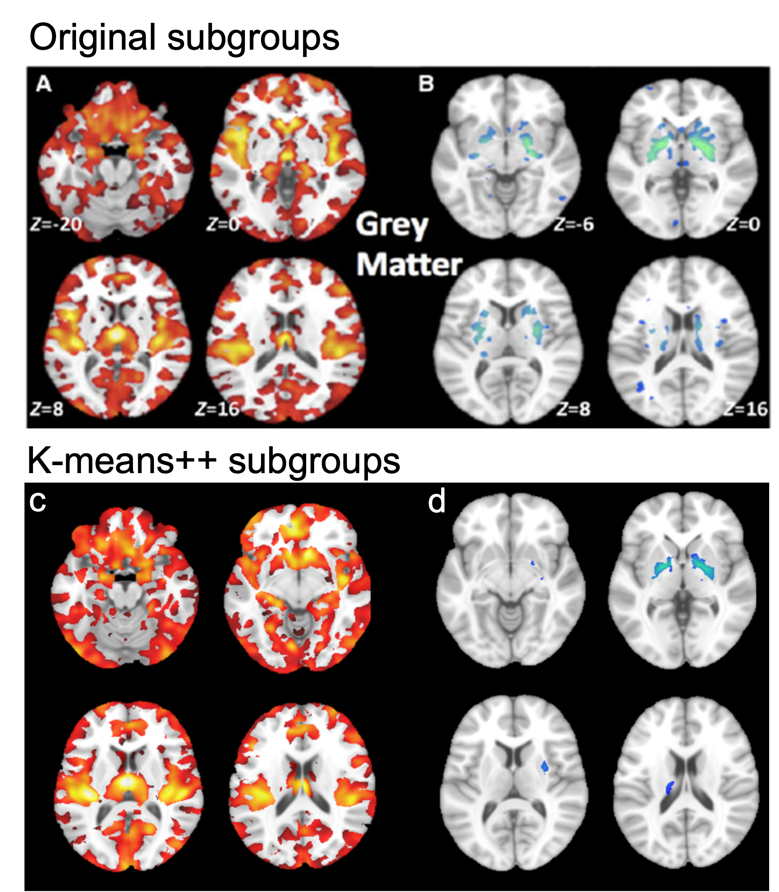


Figure 15. The original subgroups defined by Chand et al. (2020) compared with those generated through k-means++ clustering. Increases of striatal volume in SG2 are reduced in effect size and spatial extent when using k-means.

**Tables**

Table S1. Questionnaires used in each center to evaluate remission.

|  | **DSM-III/IV/DSM-V** | **ICD-10** | **PANSS** | **WHO life chart** | **SCAN** | **GAF** | **SAPS** | **SANS** |
| --- | --- | --- | --- | --- | --- | --- | --- | --- |
| **London** | x | x |  | x | x |  |  |  |
| **Melbourne** | x |  |  | x |  |  |  |  |
| **Santander** | x | x |  |  |  |  | x | x |
| **São Paulo (PSYCLASS only)** | x |  | x |  |  | x |  |  |

Notes: DSM-III/DSM-IV/DSM-V, Diagnostic and Statistical Manual III/IV/V^40^; ICD-10, International Classification of Disease-10^41^; PANSS, Positive and Negative Symptom Questionnaire^42^; WHO life chart, World Health Organisation life chart; SCAN, Schedules for Clinical Assessment in Neuropsychiatry^43^; GAF, global assessment of functioning^44^; Scale for the Assessment of Positive Symptoms ^45^; SANS, Scale for the Assessment of Negative Symptoms^46^.

Table S2. Number of remissions at each follow-up time period for each site (remission/total).

| **Site** | **1-year remission** | **3-year remission** | **5-year remission** | **10-year remission** | **Ever Remitted** |
| --- | --- | --- | --- | --- | --- |
| London | -- | -- | 51/86 | -- | 51/86 |
| Santander | 115/166 | 101/147 | -- | 19/36 | 138/172 |
| Sao Paolo | 35/95 | -- | 19/49 | -- | 42/96 |

Notes: “--”, data not available for the site.

Table S3. Description of case/control groups used in analyses

| **Variable** | **Controls** | **First-episode** | **F/Chi** | **p-value** |
| --- | --- | --- | --- | --- |
| N | 424 | 572 |  |  |
| Current Age | 27.3(6.7) | 26.2(7.0) | -- | -- |
| Gender (%M) | 239(56.4) | 368(64.3) | -- | -- |
| Marital Status (Single %) | 102(45.3) | 251(72.5) | 42.99(6) | 4.63E-10 |
| Employment Status (employed %) | 156(69.6) | 155(38.5) | 77.34(12) | 3.02E-15 |
| Highest Education  (1-4 highest) | 2.8(1.0) | 2.4(1.0) | -- | -- |
| Onset | -1.0(0.0) | 25.0(7.6) | -- | -- |
| CPZ dose | -- | 2734.6(7702.4) | -- | -- |
| GAF | -- | 50.7(32.8) | -- | -- |
| PANSS Positive | -- | 14.0(6.5) | -- | -- |
| PANSS Negative | -- | 13.8(6.5) | -- | -- |
| PANSS General | -- | 28.4(9.1) | -- | -- |
| PANSS Total | -- | 56.3(17.9) | -- | -- |

Notes: Highest education: range 1-4; PANSS: positive and negative syndrome scale; GAF: global assessment of functioning; CPZ: chlorpromazine equivalent dose; DUP: duration of untreated psychosis; MDD: major depressive disorder; Psychosis NOS: psychosis not otherwise specified; “—" indicates data unavailable for that site. The unavailability of data results in differing degrees of freedom.

Table S4. Baseline demographic, clinical and medication differences across sites in FEP.

|  | Sao Paulo (PSYCLASS) | Sao Paulo (ESNA) | Melbourne | Santander | London | F/Chi2 | p-value |
| --- | --- | --- | --- | --- | --- | --- | --- |
| N | 102 | 26 | 136 | 186 | 122 |  |  |
| Age (mean;SD) | 26.9(7.1) | 27.0(7.4) | 21.5(3.2) | 28.7(7.6) | 26.9(6.3) | 26.44(4,567) | 3.97e-20 |
| Gender (male, %) | 58(56.9) | 17(65.4) | 90(66.2) | 117(62.9) | 86(70.5) | 4.88(10) | 3.00e-01 |
| Marital Status (single, %) | 61(59.8) | -- | -- | 141(78.3) | 49(76.6) | 11.86(10) | 2.70E-03 |
| Unemployed (yes; %) | 64(62.7) | -- | -- | 77(43.0) | -- | 10.12(10) | 1.50E-03 |
| Highest Education (1-4 scale; median) | 2 | 2 | -- | 3 | 3 | 39.8(3,385) | 1.21E-08 |
| Family History (yes, %) | 26(25.5) | 2(7.7) | 12(8.8) | 19(10.3) | -- | 17.86(10) | 5.00E-04 |
| *Diagnoses* | 59(57.8) | 0(0.0) | 21(15.7) | 73(39.2) | 24(22.4) | 270.72(40) | 1.49e-41 |
| Schizophrenia (yes; %) | 59(57.8) | 0(0.0) | 21(15.4) | 73(39.2) | 24(19.7) | 74.74(10) | 2.26e-15 |
| Schizoaffective (yes; %) | 0(0.0) | 0(0.0) | 13(9.6) | 4(2.2) | 10(8.2) | 19.43(10) | 6.47e-04 |
| Schizophreniform (yes;%) | 0(0.0) | 15(57.7) | 51(37.5) | 64(34.4) | 30(24.6) | 61.66(10) | 1.30e-12 |
| Psychosis NOS (yes;%) | 0(0.0) | 9(34.6) | 11(8.1) | 15(8.1) | 15(12.3) | 33.70(10) | 8.58e-07 |
| Brief Psychotic Disorder (yes;%) | 0(0.0) | 2(7.7) | 6(4.4) | 30(16.1) | 0(0.0) | 44.06(10) | 6.23e-09 |
| Drug Induced Psychosis (yes;%) | 0(0.0) | 0(0.0) | 4(2.9) | 0(0.0) | 0(0.0) | 12.91(10) | 1.17e-02 |
| Bipolar-I (yes;%) | 25(24.5) | 0(0.0) | 18(13.2) | 0(0.0) | 14(11.5) | 49.45(10) | 4.71e-10 |
| Bipolar-II (yes;%) | 0(0.0) | 0(0.0) | 0(0.0) | 0(0.0) | 0(0.0) | -- | -- |
| MDD (yes;%) | 18(17.6) | 0(0.0) | 10(7.4) | 0(0.0) | 14(11.5) | 35.78(10) | 3.21e-07 |
| Onset Age (yrs; mean(SD)) | 26.1(7.1) | 27.0(7.4) | 20.9(3.0) | 27.8(7.7) | -- | 28.97(3,427) | 4.56e-17 |
| Duration of Illness (years; mean(SD)) | 0.8(1.0) | 0.0(0.0) | 0.6(1.0) | 0.9(1.3) | -- | 5.30(3,417) | 1.36e-03 |
| Duration of Untreated Psychosis (mths; mean(SD)) | 14.9(30.7) | -- | -- | 8.2(16.0) | 20.5(35.4) | 6.77(2,359) | 1.30e-03 |
| CPZ dose (mean(SD)) | 164.0(186.0) | -- | 165.2(130.6) | 185.2(150.9) | 11188.8(12771.3) | 77.58(3,405) | 1.14e-39 |
| GAF (mean(SD)) | -- | 32.6(11.3) | -- | 52.2(33.5) | -- | 4.72(1,190) | 3.11e-02 |
| PANSS Positive (mean(SD)) | 10.3(4.8) | 19.8(5.5) | 23.4(6.3) | 20.2(5.6) | 14.7(5.7) | 77.82(4,345) | 5.52e-47 |
| PANSS Negative (mean(SD)) | 12.3(6.0) | 17.4(8.7) | 20.4(6.7) | 10.7(4.6) | 15.5(6.0) | 26.18(4,345) | 5.69e-19 |
| PANSS General (mean(SD)) | 22.9(5.2) | 39.7(11.4) | 42.2(8.3) | 33.9(9.6) | 29.9(6.9) | 94.35(4,345) | 3.93e-54 |
| PANSS Total (mean(SD)) | 45.5(11.7) | 76.8(23.5) | 85.9(16.2) | 64.8(16.2) | 60.1(14.0) | 99.36(4,345) | 3.59e-56 |

Notes: PANSS: positive and negative syndrome scale; GAF: global assessment of functioning; CPZ: chlorpromazine equivalent dose; DUP: duration of untreated psychosis; MDD: major depressive disorder; Psychosis NOS: psychosis not otherwise specified; “—" indicates data unavailable for that site. The unavailability of data results in differing degrees of freedom.

Table S5. Analysis of differences between baseline and follow-up samples

| Variable | Baseline Only | Follow-up | T/Chi2 | p-value | Eta2/Phi |
| --- | --- | --- | --- | --- | --- |
| N | 218 | 354 |  |  |  |
| Subgroups | 96(44.0) | 121(34.2) | 7.43(8) | -- | -- |
| None | 96(44.0) | 121(34.2) | 5.57(4) | -- | -- |
| SG1 | 64(29.4) | 120(33.9) | 1.27(4) | -- | -- |
| SG2 | 44(20.2) | 74(20.9) | 0.04(4) | -- | -- |
| SG1+SG2 | 14(6.4) | 39(11.0) | 3.39(4) | -- | -- |
| Age (mean;SD) | 23.6(5.7) | 27.8(7.2) | -7.42(570) | 4.25e-13 | 0.09 |
| Gender (male, %) | 140(64.2) | 228(64.4) | 0.00(4) | -- | -- |
| Marital Status (single, %) | 22(68.8) | 229(72.9) | 0.25(4) | -- | -- |
| Unemployed (yes; %) | 14(73.7) | 127(48.5) | 4.50(4) | -- | -- |
| Highest Education (1-4 scale; mean(SD)) | 2.5(1.1) | 2.4(1.0) | 0.09(387) | -- | -- |
| Family History (yes, %) | 17(9.3) | 42(15.7) | 3.87(4) | -- | -- |
| Any Diagnosis | 31(15.3) | 146(41.5) | 52.46(16) | 4.74e-09 | 0.30 |
| Schizophrenia (yes; %) | 31(14.2) | 146(41.2) | 46.10(4) | 1.12e-11 | 0.28 |
| Schizoaffective (yes; %) | 16(7.3) | 11(3.1) | 5.37(4) | 2.05e-02 | 0.10 |
| Schizophreniform (yes;%) | 74(33.9) | 86(24.3) | 6.24(4) | 1.25e-02 | 0.10 |
| Psychosis NOS (yes;%) | 25(11.5) | 25(7.1) | 3.28(4) | -- | -- |
| Brief Psychotic Disorder (yes;%) | 12(5.5) | 26(7.3) | 0.74(4) | -- | -- |
| Drug Induced Psychosis (yes;%) | 4(1.8) | 0(0.0) | 6.54(4) | 1.05e-02 | 0.11 |
| Bipolar-I (yes;%) | 23(10.6) | 34(9.6) | 0.13(4) | -- | -- |
| Bipolar-II (yes;%) | 0(0.0) | 0(0.0) | NaN(2) | -- | -- |
| MDD (yes;%) | 18(8.3) | 24(6.8) | 0.43(4) | -- | -- |
| Onset Age (yrs; mean(SD)) | 22.5(5.2) | 27.2(7.5) | -7.24(429) | 2.05e-12 | 0.11 |
| Duration of Illness (years; mean(SD)) | 0.6(1.2) | 0.9(1.1) | -2.26(419) | -- | -- |
| Duration of Untreated Psychosis (mths; mean(SD)) | 15.4(32.0) | 12.7(25.9) | 0.55(360) | -- | -- |
| CPZ dose (mean(SD)) | 2615.6(6008.2) | 2794.1(8079.8) | -0.18(407) | -- | -- |
| GAF (mean(SD)) | 43.6(28.4) | 51.9(33.4) | -1.22(190) | -- | -- |
| PANSS Positive (mean(SD)) | 21.5(6.8) | 13.0(6.2) | 12.29(348) | 4.39e-29 | 0.31 |
| PANSS Negative (mean(SD)) | 19.0(7.1) | 13.2(6.2) | 8.17(348) | 5.68e-15 | 0.16 |
| PANSS General (mean(SD)) | 40.0(9.7) | 26.5(7.5) | 14.53(348) | 1.05e-37 | 0.39 |
| PANSS Total (mean(SD)) | 80.4(19.6) | 52.8(15.2) | 14.82(348) | 7.27e-39 | 0.40 |
| CPZ dose (Mean(SD)) | 2615.6(6008.2) | 2794.1(8079.8) | -0.18(407) | -- | -- |
| CPZ Z-norm (Mean(SD)) | 0.4(1.2) | 0.0(1.0) | 2.92(407) | 3.75e-03 | 0.03 |
| Treatment Duration (mths; mean(SD)) | 3.6(5.1) | 3.1(2.5) | 0.69(228) | -- | -- |
| Lithium (yes, %) | 17(10.9) | 10(3.7) | 8.49(4) | 3.57e-03 | 0.12 |
| Other mood stabilizers (yes, %) | 0(0.0) | 1(0.6) | 0.08(4) | -- | -- |
| Antidepressants (yes, %) | 1(5.0) | 23(8.7) | 0.33(4) | -- | -- |
| Anxiolytics (yes, %) | 11(55.0) | 140(53.0) | 0.03(4) | -- | -- |
| Antiepileptics (yes, %) | 1(16.7) | 8(8.3) | 0.49(4) | -- | -- |
| Antipsychotic class typical/atypical (typical, %) | 5(12.2) | 39(13.3) | 0.04(4) | -- | -- |
| Marijuana (yes, %) | 9(19.6) | 105(39.2) | 6.53(4) | 1.06e-02 | 0.11 |
| Other illicit (yes, %) | 45(71.4) | 66(24.8) | 49.51(4) | 1.97e-12 | 0.29 |
| Tobacco (yes, %) | 7(50.0) | 91(54.5) | 0.10(4) | -- | -- |

Table S6. Comparison of baseline demographics across subgroups in healthy control subjects.

| **Variable** | **None** | **Subgroup1** | **Subgroup2** | **S1+S2** | **FChi** | **P** | Eta2/Phi |
| --- | --- | --- | --- | --- | --- | --- | --- |
| N | 227 | 82 | 96 | 19 |  |  |  |
| Age (mean;SD) | 27.0(6.9) | 28.0(6.9) | 27.2(6.5) | 28.3(5.2) | 0.64(3,420) | -- | 0.00 |
| Gender (male, %) | 128(56.4) | 49(59.8) | 50(52.1) | 12(63.2) | 1.46(8) | -- | 0.06 |
| Marital Status (single, %) | 57(48.3) | 27(45.0) | 14(36.8) | 4(44.4) | 1.53(8) | -- | 0.06 |
| Unemployed (yes; %) | 26(26.3) | 13(24.1) | 13(40.6) | 3(37.5) | 3.39(8) | -- | 0.09 |
| Highest Education (1-4 scale; mean(SD)) | 2.9(0.9) | 2.5(1.1) | 3.0(0.9) | 2.8(1.1) | 2.79(3,245) | 0.04* | 0.18 |

* p-value significant at uncorrected family-wise significance levels

Table S7. Number of cases who remitted within each subgroup with percentage compared to non-remitters within the subgroup

| **Variable** | **N** | **None** | **Subgroup1** | **Subgroup2** | **S1+S2** | **Chi^2^** | **P-value** | **Phi** |
| --- | --- | --- | --- | --- | --- | --- | --- | --- |
| 1 year (n;%) | 261 | 47(55.3) | 48(52.7) | 41(73.2) | 14(48.3) | 7.68(8) | 0.05 | 0.15 |
| 3 year (n;%) | 147 | 29(69.0) | 29(67.4) | 29(67.4) | 14(73.7) | 0.29(8) | -- | 0.03 |
| 5 year (n;%) | 135 | 28(49.1) | 20(43.5) | 14(73.7) | 8(61.5) | 5.58(8) | -- | 0.13 |
| Ever Remitted (n;%) | 354 | 78(64.5) | 68(56.7) | 58(78.4) | 27(69.2) | 9.83(8) | 0.02 | 0.17 |

Table S8. Analysis of medication associations with brain subgroups (FDR significance across 4 comparisons)

|  | N | None | SG1 | SG2 | SG1+SG2 | F/Chi2 | p-value | Eta2/Phi | Pairwise |
| --- | --- | --- | --- | --- | --- | --- | --- | --- | --- |
| N |  | 217 | 184 | 118 | 53 |  |  |  |  |
| CPZ dose (Mean(SD)) | 409 | 2328.9 (5171.6) | 3091.5 (9094.5) | 2870.4 (9072.3) | 2961.2 (7690.0) | 0.25(3,405) | -- | -- |  |
| CPZ Z-norm (Mean(SD)) | 409 | -0.1(0.8) | 0.1(0.8) | 0.2(1.6) | 0.4(1.1) | 2.88(3,405) | 3.56e-02 | 0.02 | None<SG1+SG2 |
| Typical/atypical (typical, %) | 335 | 13(11.2) | 23(21.7) | 2(2.7) | 6(15.4) | 14.42(8) | 2.38e-03 | 0.16 | SG1>None,SG2;SG2<None,SG1+SG2 |
| Treatment Duration (mths; mean(SD)) | 230 | 3.0(2.5) | 3.3(2.6) | 2.9(2.2) | 4.1(4.8) | 1.07(3,226) | -- | -- |  |

Notes: *, non-significant at corrected levels

Table S9. Comparison of subgroups only in individuals with follow-up data.

| Variable | None | SG1 | SG2 | SG1+SG2 | F/Chi2 | p-value | Eta2/Phi |
| --- | --- | --- | --- | --- | --- | --- | --- |
| N | 217 | 184 | 118 | 53 |  |  |  |
| Site | 58(26.7) | 39(21.2) | 30(25.4) | 9(17.0) | 30.25(16) | 3.97e-04 | 0.23 |
| London | 55(25.3) | 32(17.4) | 24(20.3) | 11(20.8) | 3.87(8) | -- | -- |
| Melbourne | 58(26.7) | 39(21.2) | 30(25.4) | 9(17.0) | 3.25(8) | -- | -- |
| Santander | 58(26.7) | 54(29.3) | 52(44.1) | 22(41.5) | 13.28(8) | 4.06e-03 | 0.15 |
| SaoPaolo | 46(21.2) | 59(32.1) | 12(10.2) | 11(20.8) | 20.32(8) | 1.46e-04 | 0.19 |
| Age (mean;SD) | 26.8(7.3) | 26.0(6.6) | 26.1(7.3) | 25.0(6.1) | 1.05(3,568) | -- | -- |
| Gender (male, %) | 131(60.4) | 127(69.0) | 74(62.7) | 36(67.9) | 3.68(8) | -- | -- |
| Marital Status (single, %) | 75(64.7) | 84(70.6) | 58(81.7) | 34(85.0) | 9.95(8) | -- | -- |
| Unemployed (yes; %) | 45(49.5) | 52(52.5) | 32(55.2) | 12(36.4) | 3.34(8) | -- | -- |
| Highest Education (1-4 scale; mean(SD)) | 2.6(1.1) | 2.1(0.9) | 2.9(0.8) | 2.3(1.0) | 10.82(3,385) | 7.73e-07 | 0.08 |
| Family History (yes, %) | 22(13.7) | 20(13.2) | 10(10.6) | 7(16.7) | 1.01(8) | -- | -- |
| Any Diagnosis | 54(26.3) | 65(35.3) | 31(27.2) | 27(51.9) | 30.34(32) | -- | -- |
| Schizophrenia (yes; %) | 54(24.9) | 65(35.3) | 31(26.3) | 27(50.9) | 16.51(8) | 8.92e-04 | 0.17 |
| Schizoaffective (yes; %) | 11(5.1) | 13(7.1) | 3(2.5) | 0(0.0) | 6.18(8) | -- | -- |
| Schizophreniform (yes;%) | 62(28.6) | 47(25.5) | 37(31.4) | 14(26.4) | 1.31(8) | -- | -- |
| Psychosis NOS (yes;%) | 20(9.2) | 14(7.6) | 12(10.2) | 4(7.5) | 0.75(8) | -- | -- |
| Brief Psychotic Disorder (yes;%) | 14(6.5) | 9(4.9) | 13(11.0) | 2(3.8) | 5.27(8) | -- | -- |
| Drug Induced Psychosis (yes;%) | 1(0.5) | 2(1.1) | 0(0.0) | 1(1.9) | 2.48(8) | -- | -- |
| Bipolar-I (yes;%) | 24(11.1) | 19(10.3) | 11(9.3) | 3(5.7) | 1.47(8) | -- | -- |
| Bipolar-II (yes;%) | 0(0.0) | 0(0.0) | 0(0.0) | 0(0.0) | NaN(4) | -- | -- |
| MDD (yes;%) | 19(8.8) | 15(8.2) | 7(5.9) | 1(1.9) | 3.48(8) | -- | -- |
| Onset Age (yrs; mean(SD)) | 26.1(7.5) | 24.9(6.6) | 25.5(7.6) | 23.8(5.9) | 1.38(3,427) | -- | -- |
| Duration of Illness (years; mean(SD)) | 0.7(1.1) | 0.7(1.1) | 0.7(1.1) | 1.3(1.7) | 2.62(3,417) | -- | -- |
| Duration of Untreated Psychosis (mths; mean(SD)) | 11.3(22.7) | 14.4(28.2) | 9.3(17.3) | 20.1(41.3) | 1.72(3,358) | -- | -- |
| CPZ dose (mean(SD)) | 2328.9(5171.6) | 3091.5(9094.5) | 2870.4(9072.3) | 2961.2(7690.0) | 0.25(3,405) | -- | -- |
| GAF (mean(SD)) | 50.3(31.7) | 51.7(33.3) | 50.9(35.4) | 48.9(29.9) | 0.04(3,188) | -- | -- |
| PANSS Positive (mean(SD)) | 16.8(6.9) | 15.8(7.8) | 19.1(8.1) | 16.9(9.3) | 2.59(3,346) | -- | -- |
| PANSS Negative (mean(SD)) | 15.5(7.1) | 15.6(7.5) | 16.0(6.9) | 17.7(7.8) | 0.78(3,346) | -- | -- |
| PANSS General (mean(SD)) | 32.8(10.5) | 31.5(11.5) | 34.1(9.9) | 34.0(12.2) | 1.00(3,346) | -- | -- |
| PANSS Total (mean(SD)) | 65.3(20.7) | 62.9(23.3) | 69.2(21.2) | 68.3(25.6) | 1.28(3,346) | -- | -- |
| CPZ dose (Mean(SD)) | 2328.9(5171.6) | 3091.5(9094.5) | 2870.4(9072.3) | 2961.2(7690.0) | 0.25(3,405) | -- | -- |
| CPZ Z-norm (Mean(SD)) | -0.1(0.8) | 0.1(0.8) | 0.2(1.6) | 0.4(1.1) | 2.88(3,405) | -- | -- |
| Treatment Duration (mths; mean(SD)) | 3.0(2.5) | 3.3(2.6) | 2.9(2.2) | 4.1(4.8) | 1.07(3,226) | -- | -- |
| Lithium (yes, %) | 11(7.2) | 6(4.3) | 8(8.9) | 2(4.8) | 2.35(8) | -- | -- |
| Other mood stabilizers (yes, %) | 1(1.8) | 0(0.0) | 0(0.0) | 0(0.0) | 2.26(8) | -- | -- |
| Antidepressants (yes, %) | 7(7.6) | 9(9.0) | 3(5.0) | 5(15.6) | 3.18(8) | -- | -- |
| Anxiolytics (yes, %) | 44(47.8) | 45(45.0) | 39(65.0) | 23(71.9) | 11.60(8) | -- | -- |
| Antiepileptics (yes, %) | 1(2.8) | 6(12.8) | 0(0.0) | 2(18.2) | 4.52(8) | -- | -- |
| Antipsychotic class typical/atypical (typical, %) | 13(11.2) | 23(21.7) | 2(2.7) | 6(15.4) | 14.42(8) | 2.38e-03 | 0.16 |
| Marijuana (yes, %) | 32(30.8) | 39(34.5) | 29(45.3) | 14(42.4) | 4.31(8) | -- | -- |
| Other illicit (yes, %) | 37(33.9) | 38(34.2) | 27(37.5) | 9(24.3) | 1.94(8) | -- | -- |
| Tobacco (yes, %) | 26(46.4) | 33(64.7) | 27(51.9) | 12(54.5) | 3.74(8) | -- | -- |

Notes: PANSS: positive and negative syndrome scale; GAF: global assessment of functioning; CPZ: chlorpromazine equivalent dose; DUP: duration of untreated psychosis; MDD: major depressive disorder; Psychosis NOS: psychosis not otherwise specified; “—" indicates data unavailable for that site. The unavailability of data results in differing degrees of freedom.

Table S10. Missingness and test of Missing Completely At Random (MCAR) in the variables used in baseline and longitudinal analyses

|  | Total Missing Data | Little’s MCAR test^1^ | p-value | Category |
| --- | --- | --- | --- | --- |
| Baseline | 21% (2756) | 1375.24(616) | <0.001 | MAR |
| 1-year follow-up prediction | 10% (732) | 482.16(311) | <0.001 | MAR |
| 3-year follow-up prediction | 14% (538) | 24.49(149) | >0.05 | MCAR |
| 5-year follow-up prediction | 15% (541) | 482.64(349) | <0.001 | MAR |
| Remission at any timepoint | 14% (1326) | 984.58(568) | <0.001 | MAR |

Notes: MAR, missing at random

Table S11. K-means++ subgroup memberships compared to the original subgroup definitions in the discovery sample. Overlap was most prominent for SG1, but SG2 subgroup membership was mixed.

| Variable | SG1 | SG2 | F/Chi2 | p-value | Eta2/Phi |
| --- | --- | --- | --- | --- | --- |
| N | 157 | 150 |  |  |  |
| Original Subgroup 1 (n;%) | 136(86.6) | 56(37.3) | 79.55(4) | 4.69e-19 | 0.51 |
| Original Subgroup 2 (n;%) | 21(13.4) | 94(62.7) | 79.55(4) | 4.69e-19 | 0.51 |
| Sex (male;%) | 109(69.4) | 90(60.0) | 2.99(4) | -- | 0.10 |
| Age (mean yrs(SD)) | 30.4(7.6) | 31.5(7.0) | -1.27(305) | -- | 0.01 |
| Sites | 52(33.1) | 44(29.3) | 0.92(6) | -- | 0.05 |
| Site 1 (n;%) | 52(33.1) | 44(29.3) | 0.51(4) | -- | 0.04 |
| Site 2 (n;%) | 70(44.6) | 75(50.0) | 0.90(4) | -- | 0.05 |
| Site 3 (n;%) | 35(22.3) | 31(20.7) | 0.12(4) | -- | 0.02 |

Table 12. K-means++ subgroups in the first-episode sample: discovery sample using only patients

| Variable | SG1 | SG2 | F/Chi2 | p-value | Eta2/Phi |
| --- | --- | --- | --- | --- | --- |
| N | 362 | 205 |  |  |  |
| **Original Subgroups** | 136(37.6) | 80(39.0) | 43.95(8) | 1.54e-09 | 0.28 |
| None (n;%) | 136(37.6) | 80(39.0) | 0.12(4) | -- | -- |
| SG1 (n;%) | 144(39.8) | 38(18.5) | 27.10(4) | 1.93e-07 | 0.22 |
| SG2 (n;%) | 49(13.5) | 69(33.7) | 32.16(4) | 1.42e-08 | 0.24 |
| SG1+SG2 (n;%) | 33(9.1) | 18(8.8) | 0.02(4) | -- | -- |
| **Site** |  |  | 111.50(8) | 5.22e-24 | 0.44 |
| London (n;%) | 90(24.9) | 32(15.6) | 6.63(4) | 1.00e-02 | 0.11 |
| Melbourne (n;%) | 107(29.6) | 29(14.1) | 17.05(4) | 3.64e-05 | 0.17 |
| Santander (n;%) | 136(37.6) | 50(24.4) | 10.31(4) | 1.32e-03 | 0.13 |
| SaoPaolo (n;%) | 29(8.0) | 94(45.9) | 110.34(4) | 8.27e-26 | 0.44 |
| **Demographics** |  |  |  |  |  |
| Age (mean;SD) | 26.4(7.1) | 25.8(6.7) | 1.06(565) | -- | -- |
| Gender (male, %) | 242(66.9) | 122(59.5) | 3.07(4) | -- | -- |
| Marital Status (single, %) | 138(71.5) | 109(73.6) | 0.19(4) | -- | -- |
| Unemployed (yes; %) | 69(46.9) | 69(53.5) | 1.18(4) | -- | -- |
| Highest Education (1-4 scale; median) | 2 | 2 | 1.92(382) | -- | -- |
| Family History (yes, %) | 32(11.8) | 26(15.0) | 0.96(4) | -- | -- |
| **Any Diagnosis** |  |  | 36.27(16) | 6.44e-06 | 0.25 |
| Schizophrenia (yes; %) | 100(27.6) | 72(35.1) | 3.48(4) | -- | -- |
| Schizoaffective (yes; %) | 23(6.4) | 4(2.0) | 5.59(4) | 1.80e-02 | 0.10 |
| Schizophreniform (yes;%) | 119(32.9) | 41(20.0) | 10.71(4) | 1.07e-03 | 0.14 |
| Psychosis NOS (yes;%) | 35(9.7) | 15(7.3) | 0.90(4) | -- | -- |
| Brief Psychotic Disorder (yes;%) | 29(8.0) | 9(4.4) | 2.74(4) | -- | -- |
| Drug Induced Psychosis (yes;%) | 3(0.8) | 1(0.5) | 0.22(4) | -- | -- |
| Bipolar-I (yes;%) | 23(6.4) | 34(16.6) | 15.15(4) | 9.91e-05 | 0.16 |
| Bipolar-II (yes;%) | 0(0.0) | 0(0.0) | NaN(2) | -- | -- |
| MDD (yes;%) | 21(5.8) | 21(10.2) | 3.77(4) | -- | -- |
| **Illness and Symptoms** |  |  |  |  |  |
| Onset Age (yrs; mean(SD)) | 25.5(7.3) | 25.1(6.8) | 0.59(424) | -- | -- |
| Duration of Illness (years; mean(SD)) | 0.8(1.3) | 0.7(0.8) | 1.21(414) | -- | -- |
| Duration of Untreated Psychosis (mths; mean(SD)) | 12.2(24.6) | 11.9(22.9) | 0.11(355) | -- | -- |
| GAF (mean(SD)) | 50.2(33.4) | 52.1(31.6) | -0.37(190) | -- | -- |
| PANSS Positive (mean(SD)) | 18.8(7.2) | 14.6(7.7) | 5.20(343) | 3.48e-07 | 0.07 |
| PANSS Negative (mean(SD)) | 17.3(7.4) | 14.0(6.7) | 4.27(343) | 2.57e-05 | 0.05 |
| PANSS General (mean(SD)) | 35.8(10.6) | 28.7(10.1) | 6.23(343) | 1.38e-09 | 0.10 |
| PANSS Total (mean(SD)) | 71.8(21.1) | 57.3(20.9) | 6.37(343) | 6.21e-10 | 0.11 |
| **Treatments** |  |  |  |  |  |
| CPZ Z-norm (Mean(SD)) | 0.1(1.1) | -0.0(0.9) | 1.57(402) | -- | -- |
| Treatment Duration (mths; mean(SD)) | 2.8(1.8) | 3.8(3.8) | -2.88(227) | 4.32e-03 | 0.04 |
| Lithium (yes, %) | 11(4.2) | 16(10.0) | 5.43(4) | 1.98e-02 | 0.10 |
| Other mood stabilizers (yes, %) | 1(0.8) | 0(0.0) | 0.38(4) | -- | -- |
| Antidepressants (yes, %) | 5(3.4) | 17(13.0) | 8.81(4) | 2.99e-03 | 0.12 |
| Anxiolytics (yes, %) | 104(70.3) | 46(35.1) | 34.55(4) | 4.15e-09 | 0.25 |
| Antiepileptics (yes, %) | 2(12.5) | 7(8.6) | 0.24(4) | -- | -- |
| Antipsychotic class typical/atypical (typical, %) | 10(4.9) | 31(24.6) | 27.98(4) | 1.23e-07 | 0.22 |

Notes: PANSS, positive and negative syndrome scale; GAF, global assessment of functioning; CPZ, chlorpromazine equivalent dose; MDD, major depressive disorder; Psychosis NOS, Psychosis not otherwise specified. Results corrected for multiple comparisons at a false-discovery rate (FDR)<0.05. ANOVA used for continuous variables, Chi^2^ for binary, and Kruskall-Wallis for education variable

Table S13. K-means++ subgroups compared across remission timepoints: discovery using only patients

| Variable | SG1 | SG2 | Chi2 | p-value | Phi |
| --- | --- | --- | --- | --- | --- |
| N | 205 | 144 |  |  |  |
| 1-year remission (n;%) | 82(62.1) | 68(54.8) | 1.40(4) | P=0.2 | 0.06 |
| 3-year remission (n;%) | 70(67.3) | 31(72.1) | 0.32(4) | P=0.5 | 0.03 |
| 5-year remission (n;%) | 40(53.3) | 30(50.8) | 0.08(4) | P=0.7 | 0.02 |
| 10-year remission (n;%) | 10(50.0) | 9(56.2) | 0.14(4) | P=0.7 | 0.02 |
| Ever Remitted (n;%) | 136(66.3) | 95(66.0) | 0.01(4) | P=0.9 | 0.00 |

Table S14. K-means++ subgroups in the first-episode sample: discovery sample using patients and healthy controls

| Variable | SG1 | SG2 | F/Chi2 | p-value | Eta2/Phi |
| --- | --- | --- | --- | --- | --- |
| N | 632 | 274 |  |  |  |
| **Original Subgroups** |  |  | 93.33(8) | 4.22e-20 | 0.32 |
| None (n;%) | 297(47.0) | 108(39.4) | 4.44(4) | -- | -- |
| SG1 (n;%) | 193(30.5) | 31(11.3) | 37.95(4) | 7.24e-10 | 0.20 |
| SG2 (n;%) | 94(14.9) | 116(42.3) | 80.95(4) | 2.31e-19 | 0.30 |
| SG1+SG2 (n;%) | 48(7.6) | 19(6.9) | 0.12(4) | -- | -- |
| **Site** |  |  | 91.51(8) | 1.04e-19 | 0.32 |
| London (n;%) | 159(25.2) | 51(18.6) | 4.60(4) | -- | -- |
| Melbourne (n;%) | 163(25.8) | 47(17.2) | 8.01(4) | 4.65e-03 | 0.09 |
| Santander (n;%) | 230(36.4) | 64(23.4) | 14.82(4) | 1.19e-04 | 0.13 |
| SaoPaolo (n;%) | 80(12.7) | 112(40.9) | 91.13(4) | 1.35e-21 | 0.32 |
| **Demographics** |  |  |  |  |  |
| Age (mean;SD) | 26.5(6.9) | 26.2(6.4) | 0.48(904) | -- | -- |
| Gender (male, %) | 393(62.2) | 162(59.1) | 0.75(4) | -- | -- |
| Marital Status (single, %) | 199(61.8) | 107(67.3) | 1.39(4) | -- | -- |
| Unemployed (yes; %) | 96(39.2) | 70(50.4) | 4.51(4) | -- | -- |
| Highest Education (1-4 scale; median) | 2 | 2 | 1.56(557) | -- | -- |
| Family History (yes, %) | 34(10.0) | 24(13.6) | 1.48(4) | -- | -- |
| **Any Diagnosis** | 105(27.9) | 67(38.5) | 38.49(16) | 2.44e-06 | 0.21 |
| Schizophrenia (yes; %) | 105(16.6) | 67(24.5) | 7.64(4) | 5.72e-03 | 0.09 |
| Schizoaffective (yes; %) | 23(3.6) | 4(1.5) | 3.14(4) | -- | -- |
| Schizophreniform (yes;%) | 127(20.1) | 33(12.0) | 8.52(4) | 3.51e-03 | 0.10 |
| Psychosis NOS (yes;%) | 38(6.0) | 12(4.4) | 0.98(4) | -- | -- |
| Brief Psychotic Disorder (yes;%) | 31(4.9) | 7(2.6) | 2.63(4) | -- | -- |
| Drug Induced Psychosis (yes;%) | 3(0.5) | 1(0.4) | 0.05(4) | -- | -- |
| Bipolar-I (yes;%) | 26(4.1) | 31(11.3) | 16.81(4) | 4.14e-05 | 0.14 |
| Bipolar-II (yes;%) | 0(0.0) | 0(0.0) | NaN(2) | -- | -- |
| MDD (yes;%) | 23(3.6) | 19(6.9) | 4.69(4) | -- | -- |
| **Illness and Symptoms** |  |  |  |  |  |
| Onset Age (yrs; mean(SD)) | 25.5(7.3) | 25.1(6.8) | 0.46(424) | -- | -- |
| Duration of Illness (years; mean(SD)) | 0.8(1.2) | 0.7(0.8) | 0.59(414) | -- | -- |
| Duration of Untreated Psychosis (mths; mean(SD)) | 12.1(24.7) | 12.1(22.5) | -0.03(355) | -- | -- |
| GAF (mean(SD)) | 51.6(33.5) | 48.1(30.8) | 0.65(190) | -- | -- |
| PANSS Positive (mean(SD)) | 18.6(7.3) | 14.4(7.7) | 5.08(343) | 6.12e-07 | 0.07 |
| PANSS Negative (mean(SD)) | 17.2(7.4) | 13.8(6.6) | 4.35(343) | 1.76e-05 | 0.05 |
| PANSS General (mean(SD)) | 35.4(10.6) | 28.6(10.2) | 5.84(343) | 1.23e-08 | 0.09 |
| PANSS Total (mean(SD)) | 71.1(21.2) | 56.8(20.8) | 6.16(343) | 2.04e-09 | 0.10 |
| **Treatments** |  |  |  |  |  |
| CPZ Z-norm (Mean(SD)) | 0.0(1.0) | -0.0(0.9) | 0.67(474) | -- | -- |
| Treatment Duration (mths; mean(SD)) | 2.7(1.8) | 4.0(3.9) | -3.28(227) | 1.20e-03 | 0.05 |
| Lithium (yes, %) | 12(3.7) | 15(9.0) | 6.13(4) | 1.33e-02 | 0.08 |
| Other mood stabilizers (yes, %) | 1(0.7) | 0(0.0) | 0.31(4) | -- | -- |
| Antidepressants (yes, %) | 6(3.8) | 16(13.4) | 8.83(4) | 2.96e-03 | 0.10 |
| Anxiolytics (yes, %) | 109(68.1) | 41(34.5) | 31.12(4) | 2.42e-08 | 0.19 |
| Antiepileptics (yes, %) | 3(14.3) | 6(7.9) | 0.80(4) | -- | -- |
| Antipsychotic class typical/atypical (typical, %) | 12(5.5) | 29(26.1) | 29.05(4) | 7.06e-08 | 0.18 |

Notes: PANSS, positive and negative syndrome scale; GAF, global assessment of functioning; CPZ, chlorpromazine equivalent dose; MDD, major depressive disorder; Psychosis NOS, Psychosis not otherwise specified. Results corrected for multiple comparisons at a false-discovery rate (FDR)<0.05. ANOVA used for continuous variables, Chi^2^ for binary, and Kruskall-Wallis for education variable

Table S15. K-means++ subgroups compared across remission timepoints: discovery using patients and healthy controls

| Variable | SG1 | SG2 | Chi2 | p-value | Phi |
| --- | --- | --- | --- | --- | --- |
| N | 218 | 131 |  |  |  |
| 1-year remission (n;%) | 90(63.4) | 60(52.6) | 3.01(4) | P=0.08 | 0.09 |
| 3-year remission (n;%) | 75(68.8) | 26(68.4) | 0.00(4) | P=0.9 | 0.00 |
| 5-year remission (n;%) | 43(53.8) | 27(50.0) | 0.18(4) | P=0.6 | 0.02 |
| 10-year remission (n;%) | 11(52.4) | 8(53.3) | 0.00(4) | P=0.9 | 0.00 |
| Ever Remitted (n;%) | 146(67.0) | 85(64.9) | 0.16(4) | P=0.6 | 0.02 |

## **References**

1. Chand GB, Dwyer DB, Erus G, Sotiras A, Varol E, Srinivasan D *et al.* Two distinct neuroanatomical subtypes of schizophrenia revealed using machine learning. *Brain* 2020; **143**(3)**:** 1027-1038.

2. Rosa P, Zanetti M, Duran F, Santos L, Menezes P, Scazufca M *et al.* What determines continuing grey matter changes in first-episode schizophrenia and affective psychosis? *Psychological Medicine* 2015; **45**(4)**:** 817-828.

3. Schaufelberger MS, Duran FL, Lappin JM, Scazufca M, Amaro E, Jr., Leite CC *et al.* Grey matter abnormalities in Brazilians with first-episode psychosis. *Br J Psychiatry Suppl* 2007; **51:** s117-122.

4. First MB, Gibbon M. The structured clinical interview for DSM-IV axis I disorders (SCID-I) and the structured clinical interview for DSM-IV axis II disorders (SCID-II). 2004.

5. Bebbington P, Nayani T. The psychosis screening questionnaire. *International Journal of methods in Psychiatric research* 1995.

6. Serpa M, Doshi J, Erus G, Chaim-Avancini T, Cavallet M, Van De Bilt M *et al.* State-dependent microstructural white matter changes in drug-naive patients with first-episode psychosis. *Psychological Medicine* 2017; **47**(15)**:** 2613-2627.

7. Vieira S, Gong Q, Scarpazza C, Lui S, Huang X, Crespo-Facorro B *et al.* Neuroanatomical abnormalities in first-episode psychosis across independent samples: a multi-centre mega-analysis. *Psychological medicine* 2021; **51**(2)**:** 340-350.

8. Andreasen NC, Carpenter WT, Jr., Kane JM, Lasser RA, Marder SR, Weinberger DR. Remission in schizophrenia: proposed criteria and rationale for consensus. *Am J Psychiatry* 2005; **162**(3)**:** 441-449.

9. Velakoulis D, Pantelis C, McGorry PD, Dudgeon P, Brewer W, Cook M *et al.* Hippocampal volume in first-episode psychoses and chronic schizophrenia: a high-resolution magnetic resonance imaging study. *Archives of general psychiatry* 1999; **56**(2)**:** 133-141.

10. Tustison NJ, Avants BB, Cook PA, Zheng Y, Egan A, Yushkevich PA *et al.* N4ITK: improved N3 bias correction. *IEEE Trans Med Imaging* 2010; **29**(6)**:** 1310-1320.

11. Doshi J, Erus G, Ou Y, Resnick SM, Gur RC, Gur RE *et al.* MUSE: MUlti-atlas region Segmentation utilizing Ensembles of registration algorithms and parameters, and locally optimal atlas selection. *Neuroimage* 2016; **127:** 186-195.

12. Davatzikos C, Genc A, Xu D, Resnick SM. Voxel-based morphometry using the RAVENS maps: methods and validation using simulated longitudinal atrophy. *Neuroimage* 2001; **14**(6)**:** 1361-1369.

13. Ou Y, Sotiras A, Paragios N, Davatzikos C. DRAMMS: Deformable registration via attribute matching and mutual-saliency weighting. *Med Image Anal* 2011; **15**(4)**:** 622-639.

14. Srinivasan D, Erus G, Doshi J, Wolk DA, Shou H, Habes M *et al.* A comparison of Freesurfer and multi-atlas MUSE for brain anatomy segmentation: Findings about size and age bias, and inter-scanner stability in multi-site aging studies. *Neuroimage* 2020; **223:** 117248.

15. Chand GB, Singhal P, Dwyer DB, Wen J, Erus G, Doshi J *et al.* Schizophrenia Imaging Signatures and Their Associations With Cognition, Psychopathology, and Genetics in the General Population. *Am J Psychiatry* 2022**:** appiajp21070686.

16. Varol E, Sotiras A, Davatzikos C. HYDRA: Revealing heterogeneity of imaging and genetic patterns through a multiple max-margin discriminative analysis framework. *Neuroimage* 2016; **145(Pt B):** 346-364.

17. Kulesza A, Taskar B. Determinantal point processes for machine learning. *arXiv preprint arXiv:12076083* 2012.

18. Varol E, Sotiras A, Davatzikos C. MIDAS: Regionally linear multivariate discriminative statistical mapping. *Neuroimage* 2018; **174:** 111-126.

19. Satterthwaite TD, Wolf DH, Calkins ME, Vandekar SN, Erus G, Ruparel K *et al.* Structural brain abnormalities in youth with psychosis spectrum symptoms. *JAMA Psychiatry* 2016; **73**(5)**:** 515-524.

20. Gong Q, Scarpazza C, Dai J, He M, Xu X, Shi Y *et al.* A transdiagnostic neuroanatomical signature of psychiatric illness. *Neuropsychopharmacology* 2019; **44:** 869-875.

21. Luo Q, Chen Q, Wang W, Desrivières S, Quinlan EB, Jia T *et al.* Association of a schizophrenia-risk nonsynonymous variant with putamen volume in adolescents: a voxelwise and genome-wide association study. *JAMA Psychiatry* 2019; **76(4):** 435-445.

22. Zhang T, Koutsouleris N, Meisenzahl E, Davatzikos C. Heterogeneity of structural brain changes in subtypes of schizophrenia revealed using magnetic resonance imaging pattern analysis. *Schizophr Bull* 2015; **41**(1)**:** 74-84.

23. Zhao Q, Cao H, Zhang W, Li S, Xiao Y, Tamminga CA *et al.* A subtype of institutionalized patients with schizophrenia characterized by pronounced subcortical and cognitive deficits. *Neuropsychopharmacology* 2022; **47**(12)**:** 2024-2032.

24. Allswede DM, Cannon TD. Prenatal inflammation and risk for schizophrenia: A role for immune proteins in neurodevelopment. *Dev Psychopathol* 2018; **30**(3)**:** 1157-1178.

25. Howes OD, McCutcheon R. Inflammation and the neural diathesis-stress hypothesis of schizophrenia: a reconceptualization. *Transl Psychiatry* 2017; **7**(2)**:** e1024.

26. Howes O, McCutcheon R, Stone J. Glutamate and dopamine in schizophrenia: an update for the 21st century. *J Psychopharmacol* 2015; **29**(2)**:** 97-115.

27. Uhlhaas PJ, Singer W. Oscillations and neuronal dynamics in schizophrenia: the search for basic symptoms and translational opportunities. *Biol Psychiatry* 2015; **77**(12)**:** 1001-1009.

28. Howes OD, Montgomery AJ, Asselin MC, Murray RM, Valli I, Tabraham P *et al.* Elevated striatal dopamine function linked to prodromal signs of schizophrenia. *Arch Gen Psychiatry* 2009; **66**(1)**:** 13-20.

29. Cannon TD. How schizophrenia develops: cognitive and brain mechanisms underlying onset of psychosis. *Trends Cogn Sci* 2015; **19**(12)**:** 744-756.

30. Fusar-Poli P, Meyer-Lindenberg A. Striatal presynaptic dopamine in schizophrenia, part II: meta-analysis of [(18)F/(11)C]-DOPA PET studies. *Schizophr Bull* 2013; **39**(1)**:** 33-42.

31. Okada N, Yahata N, Koshiyama D, Morita K, Sawada K, Kanata S *et al.* Abnormal asymmetries in subcortical brain volume in early adolescents with subclinical psychotic experiences. *Transl Psychiatry* 2018; **8**(1)**:** 254.

32. Zhang W, Deng W, Yao L, Xiao Y, Li F, Liu J *et al.* Brain structural abnormalities in a group of never-medicated patients with long-term schizophrenia. *Am J Psychiatry* 2015; **172**(10)**:** 995-1003.

33. Oertel-Knöchel V, Knöchel C, Matura S, Rotarska-Jagiela A, Magerkurth J, Prvulovic D *et al.* Cortical–basal ganglia imbalance in schizophrenia patients and unaffected first-degree relatives. *Schizophr Res* 2012; **138**(2-3)**:** 120-127.

34. Chemerinski E, Byne W, Kolaitis JC, Glanton CF, Canfield EL, Newmark RE *et al.* Larger putamen size in antipsychotic-naive individuals with schizotypal personality disorder. *Schizophr Res* 2013; **143**(1)**:** 158-164.

35. Fung G, Cheung C, Chen E, Lam C, Chiu C, Law CW *et al.* MRI predicts remission at 1 year in first-episode schizophrenia in females with larger striato-thalamic volumes. *Neuropsychobiology* 2014; **69**(4)**:** 243-248.

36. Chua SE, Deng Y, Chen EY, Law CW, Chiu CP, Cheung C *et al.* Early striatal hypertrophy in first-episode psychosis within 3 weeks of initiating antipsychotic drug treatment. *Psychol Med* 2009; **39**(5)**:** 793-800.

37. Williams OOF, Coppolino M, George SR, Perreault ML. Sex Differences in Dopamine Receptors and Relevance to Neuropsychiatric Disorders. *Brain Sci* 2021; **11**(9).

38. Eugene AR, Masiak J. A pharmacodynamic modelling and simulation study identifying gender differences of daily olanzapine dose and dopamine D2-receptor occupancy. *Nord J Psychiatry* 2017; **71**(6)**:** 417-424.

39. Zheng ZS, Monti MM. Cortical and thalamic connections of the human globus pallidus: Implications for disorders of consciousness. *Front Neuroanat* 2022; **16:** 960439.

40. Guze SB. Diagnostic and statistical manual of mental disorders, (DSM-IV). *American Journal of Psychiatry* 1995; **152**(8)**:** 1228-1228.

41. Organization WH. *The ICD-10 classification of mental and behavioural disorders: clinical descriptions and diagnostic guidelines*. World Health Organization1992.

42. Kay SR, Opler LA, Lindenmayer J-P. The positive and negative syndrome scale (PANSS): rationale and standardisation. *The British Journal of Psychiatry* 1989; **155**(S7)**:** 59-65.

43. Wing JK, Babor T, Brugha T, Burke J, Cooper JE, Giel R *et al.* SCAN: schedules fonr clinical assessment in neuropsychiatry. *Archives of general psychiatry* 1990; **47**(6)**:** 589-593.

44. Hall RC. Global assessment of functioning: a modified scale. *Psychosomatics* 1995; **36**(3)**:** 267-275.

45. Andreasen NC. Methods for assessing positive and negative symptoms. 1990.

46. Andreasen NC. The Scale for the Assessment of Negative Symptoms (SANS): conceptual and theoretical foundations. *The British journal of psychiatry* 1989; **155**(S7)**:** 49-52.
